# Effect of Late Second to Early Third Trimester of Pregnancy on the Activity of Renal Organic Anion Transporters (OAT1 and OAT3): A Biomarker Study

**DOI:** 10.1002/cpt.70321

**Published:** 2026-05-11

**Authors:** Aarzoo Thakur, Jashvant D. Unadkat, Emily E. Fay, Nina Isoherranen, Mary F. Hebert

**Affiliations:** ^1^ Department of Pharmaceutics, School of Pharmacy University of Washington Seattle Washington USA; ^2^ Department of Obstetrics and Gynecology, School of Medicine University of Washington Seattle Washington USA; ^3^ Department of Pharmacy, School of Pharmacy University of Washington Seattle Washington USA

## Abstract

Pregnant individuals take drugs throughout pregnancy and many of these drugs (e.g., antivirals, antibiotics) are eliminated by renal organic anion transporters (OAT) 1 and OAT3. *In vivo* studies with OAT1/3 substrate drugs suggest that pregnancy increases renal OAT1/3 activities by 1.5‐ to 1.8‐fold. However, data from these *in vivo* studies are only available for some trimesters. Various endogenous metabolites (e.g., pyridoxic acid, glycochenodeoxycholate‐3‐sulfate (GCDCA‐S)) have been identified as substrates of renal OAT1/3 and can potentially be utilized as phenotypic markers to study modulation of OAT1/3 activities across all trimesters of pregnancy. In this proof‐of‐concept study, we quantified the effect of late second to early third trimester (T2–T3) of pregnancy on the plasma concentration and renal clearance (CL_R_) of OAT1/3 biomarkers. Targeted metabolomic analysis of paired plasma and urine samples (*n* = 46 pregnant women), collected during T2–T3 (25–29 weeks of gestation) and > 3 months postpartum (PP), was conducted. Average plasma concentrations of most OAT1/3 biomarkers were 27–43% lower in T2–T3 vs. PP. The CL_R_ ratios (T2–T3/PP) of biomarkers were 1.3–1.6, except for GCDCA‐S and kynurenine where the ratios were ~5. The net renal secretory clearance and net unbound secretory clearance ratios of pyridoxic acid, p‐cresol sulfate, p‐cresol glucuronide, 3‐indoxyl sulfate, and GCDCA‐S were similar to the values listed above. Overall, OAT1/3 activity (as measured by endogenous biomarkers) is increased in T2–T3, consistent with available *in vivo* OAT1/3 substrate drug data. The studied biomarkers can be utilized in the future to quantify pregnancy‐related changes in OAT1/3 transport activities across all trimesters.

**Study Highlights**

**WHAT IS THE CURRENT KNOWLEDGE ON THE TOPIC?**

Pregnancy‐related changes lead to a 1.3‐ to 1.8‐fold increase in the renal clearance (CL_R_) of drugs excreted by renal organic anion transporters (OAT)1 and OAT3. The available data are limited due to logistical challenges associated with such *in vivo* studies. Biomarkers such as pyridoxic acid, glycochenodeoxycholate‐3‐sulfate (GCDCA‐S), kynurenic acid, and taurine are known substrates of OAT1/3 and have been utilized to predict renal OAT1/3‐mediated drug–drug interactions in healthy adult non‐pregnant individuals.

**WHAT QUESTION DID THIS STUDY ADDRESS?**

This proof‐of‐concept study evaluated the utility of OAT1/3 biomarkers to predict renal OAT1/3 transport activities in late second to early third trimester of pregnancy (T2–T3). We quantified the plasma concentrations and renal clearances (CL_R_, net CL_sec_, and net CL_sec,u_) of OAT1/3 biomarkers in T2–T3 and postpartum (PP). The net CL_sec,u_ of OAT1/3 biomarkers in T2–T3 was 1.2‐ to 1.4‐fold of PP, indicating that pregnancy‐related changes lead to increased renal OAT1/3 activity.

**WHAT DOES THIS STUDY ADD TO OUR KNOWLEDGE?**

The current study utilized multiple biomarkers from different metabolic pathways to estimate the induction of renal OAT1/3 in T2–T3. These biomarkers were verified by comparing the results with previous probe drug data.

**HOW MIGHT THIS CHANGE CLINICAL PHARMACOLOGY OR TRANSLATIONAL SCIENCE?**

The studied OAT1/3 biomarkers can be utilized to evaluate trimester‐dependent induction in renal OAT1/3 activity to inform dosing regimens of OAT1/3 substrates (e.g. antivirals, antibiotics) throughout pregnancy.

Pregnant individuals take drugs throughout pregnancy due to pre‐existing conditions (diabetes, hypertension, infections) or those caused by pregnancy.^1^ Around 70% of pregnant individuals take at least one drug, while 31% take at least five drugs during pregnancy.^1^, ^2^, ^3^ These drugs include, but are not limited to, antibiotics, antivirals, anti‐depressants, corticosteroids, and analgesics. Pregnancy can affect renal clearance (CL_R_) of drugs by one or more of the following mechanisms. Renal plasma flow increases from 0.534 L/min in pre‐pregnancy to 0.833 L/min in the first trimester (T1),^4^ resulting in a 30–50% increase in glomerular filtration rate (GFR) and therefore a possible increase in filtration clearance (CL_filtration_) and secretory clearance (CL_sec_) of drugs.^4^, ^5^, ^6^, ^7^ A decrease in blood concentrations of drug‐binding proteins like albumin and α_1_‐acid glycoprotein during pregnancy can lead to an increase in the free fraction and thereby CL_filtration_ and CL_sec_ of plasma protein‐bound drugs.^8^ Likewise, induction/repression of renal drug transporter activity can alter CL_sec_ of drugs during pregnancy. For example, CL_sec_ of amoxicillin (substrate of renal organic anion transporters, OAT1 and OAT3) is > 50% higher in second and third trimesters (T2 and T3) vs. postpartum (PP).^9^

OAT1 and OAT3 are localized on the basolateral membrane of the renal proximal tubular cells and work in tandem with multidrug resistance‐associated proteins, localized on the apical membrane, to transport drugs from blood into urine.^10^ OAT1/3‐mediated basolateral uptake is often considered the rate‐determining step for CL_sec_ of substrate drugs into urine. Drugs such as penicillin, furosemide, tenofovir, adefovir, famotidine, amoxicillin, oseltamivir, pravastatin, cefazolin, cefradine, ciprofloxacin, cefonicid, and cefaclor are substrates of OAT1/3.^10^, ^11^ *In vivo* studies of CL_R_ of these drugs suggest that pregnancy induces the activities of renal OATs by 1.5‐ to 2‐fold.^7^, ^9^, ^12^, ^13^, ^14^, ^15^ However, such data are sparse, especially in T1.

Pregnancy‐related induction of renal transport activity of drugs extensively cleared by renal excretion could lead to sub‐therapeutic plasma concentrations, necessitating dose adjustments during pregnancy. Conducting *in vivo* pharmacokinetic studies in pregnant individuals is challenging due to logistical concerns, thus limiting availability of such data for early gestation (e.g., T1). This necessitates the utility of alternate approaches to predict drug pharmacokinetics during pregnancy. One such approach is to quantify endogenous substrates as non‐invasive reporters of longitudinal changes in renal drug transporter activity during pregnancy. Shen *et al*.^16^ discovered pyridoxic acid, a catabolic end‐product of Vitamin B6, as an endogenous substrate and phenotypic biomarker of OAT1/3 activity. Pyridoxic acid is utilized as an established and validated OAT1/3 biomarker to assess drug–drug interaction risk.^16^, ^17^, ^18^ Similarly, *in vivo* studies by other groups have identified metabolites from different biological pathways as putative biomarkers of OAT1/3 activity, including kynurenic acid, taurine, glycochenodeoxycholate‐3‐sulfate (GCDCA‐S), phenylacetylglutamine, 1,7‐dimethyluric acid, androsterone glucuronide, kynurenine, and p‐cresol glucuronide.^17^, ^19^, ^20^, ^21^, ^22^

Here, we hypothesized that changes in net CL_sec_ of OAT1/3 biomarkers can be utilized to quantify pregnancy‐related induction in OAT1/3 activity. In this proof‐of‐concept study, we tested this hypothesis by quantifying the effect of late second to early third trimester (T2–T3) on the CL_R_ and net CL_sec_ of OAT1/3 biomarkers in 46 pregnant women. We carried out targeted metabolomics of paired plasma and urine samples collected during T2–T3 (25–29 weeks) and PP (≥ 3 months). Using the plasma and urine data, CL_R_, net CL_sec_, and net unbound CL_sec_ (CL_sec,u_) values of OAT1/3 biomarkers were determined. The obtained changes in net CL_sec_ and net CL_sec,u_ of OAT1/3 biomarkers were compared with the published data of OAT1/3 substrate drugs.

## MATERIALS AND METHODS

### Materials

Detailed material list is provided in the **Supplementary file**.

### *In vivo* study design

**Figure 1:**
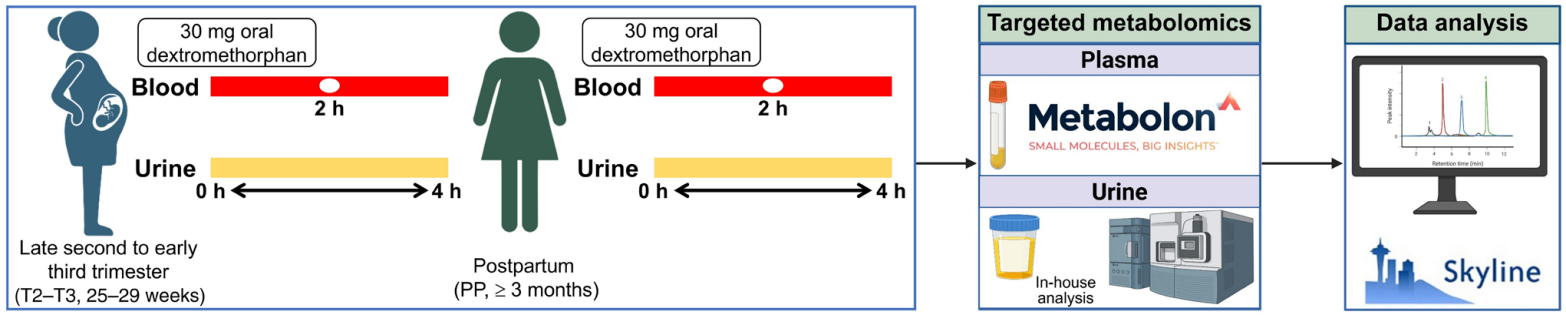
Workflow of the *in vivo* study and metabolomics assay. Paired *in vivo* study design involving 46 pregnant women and stepwise workflow of the metabolomic analysis. PP, postpartum; T2–T3, late second to early third trimester.

Details pertaining to the study design and eligibility criteria are provided elsewhere.^23^ Briefly, 46 healthy, pregnant women with singleton pregnancies, between the age of 18 and 50 years, were enrolled (**Figure** **1**). Women were excluded for fever, cough, known kidney or liver disease, diabetes, body mass index (BMI) > 30 kg/m^2^, and psychiatric illnesses requiring medication. Because the original study was designed to evaluate the effect of pregnancy on the activity of hepatic cytochrome P450 (CYP) 2D6 enzyme, participants completed three study days and were administered 30 mg dextromethorphan (CYP2D6 probe drug). In this substudy, we conducted metabolomic analysis of the plasma and urine samples collected on study days 1 (T2–T3; 25–29 weeks gestation) and 3 (≥ 3 months PP). Following 30 mg dextromethorphan administration, urine was collected over a 4‐h period (urine volume was recorded). At 2 h, a single midpoint blood sample was collected in EDTA tubes, placed on ice, and centrifuged at 4°C to separate plasma. Targeted metabolomics of the collected plasma and urine samples were conducted by Metabolon Inc. (Morrisville, NC) and in‐house, respectively (**Figure** **1**).

### Preparation and metabolomics analysis of plasma samples

Targeted metabolomic analysis of the plasma samples was conducted by Metabolon Inc. (Morrisville, NC) as described previously.^23^, ^24^

### In‐house LC‐MS/MS‐based quantification of biomarkers in urine samples

Of the > 40 clinical OAT1/3 biomarkers quantified in plasma, 10 biomarkers were selected based on the following criteria: (i) multiple *in vivo* or preclinical studies reporting them as OAT1/3 substrates; (ii) *in vitro* data showing them as substrates of OAT1/3; and (iii) availability of their standards. Details about the processing of urine samples and development and validation of an LC‐MS/MS‐based targeted metabolomics method for analysis of urine samples are provided in the **Supplementary file**. The processed urine samples (including for method validation) were analyzed using the Waters ACQUITY ultra‐performance liquid chromatography (UPLC) coupled with the Xevo TQ‐S instrument containing an electrospray ionization source. A detailed description of the LC‐MS/MS parameters is provided in the **Supplementary file** and **Tables** **S1** **and** **S2**.

### Quantification of the fraction unbound of the biomarkers in plasma (f_u,plasma_)

To conserve resources, 12 participants were randomly chosen from the 46 study participants for quantification of the fraction unbound of the biomarker in plasma (f_u,plasma_) using Thermo Scientific™ RED (rapid equilibrium dialysis) plate. Method details are provided in the **Supplementary file**.

### Data and pharmacokinetic analysis

LC‐MS/MS data obtained from LC‐MS analysis of urine and fraction unbound samples were analyzed using Skyline 25.1.^25^ Details of pharmacokinetic analysis are provided in the **Supplementary file** and **Table** **S3**. The log‐transformed plasma concentrations in T2–T3 and PP were compared using the paired *t*‐test, followed by estimation of q‐value to adjust type one error rate for multiple comparisons (*P*‐value < 0.05 and *q*‐value < 0.01 were considered statistically significant, as detailed in Enthoven *et al*.^23^). The log‐transformed amount excreted unchanged in the urine, CL_R_, net CL_sec_, net CL_sec,u_, and f_u,plasma_ in T2–T3 and PP was compared using the paired *t*‐test (GraphPad Prism; v8.4.3; San Diego, CA), with *P*‐value less than 0.05 as statistically significant. In the pregnancy arm, the gestational week range is small (25–29 weeks). Therefore, T2 and T3 were grouped together as T2–T3 for analysis. The correlation between net CL_sec,u_ of pyridoxic acid, GCDCA‐S, p‐cresol sulfate, p‐cresol glucuronide, and 3‐indoxyl sulfate in T2–T3 and PP was determined using the Pearson correlation test.

### Ethics statement

The *in vivo* study protocol and the informed consent forms were reviewed and approved by the University of Washington Institutional Review Board. The study was registered with the ClinicalTrials.gov database (NCT03117660). Participants provided written informed consent to participate in the study.

## RESULTS

### Effect of late second to early third trimester (T2–T3) on plasma concentration and renal clearance (CL_R_ ) of creatinine

The average plasma concentration of creatinine was 19% lower in T2–T3 vs. PP (adjusted *P*‐value < 0.05, **Figure** **S1**). Creatinine CL_R_ in T2–T3 was 1.3‐fold of that in PP (*P*‐value < 0.05, **Figure** **S1**, **Table** **1**).

**Table 1:** Geometric means (90% confidence interval) of plasma concentrations and renal clearance of creatinine and established and putative OAT1/3 biomarkers in pregnant women (*n* = 46) during late second to early third trimester (T2–T3) and postpartum (PP)

Metabolite	Plasma concentration ratio (T2–T3/PP)	Estimated CL_R_ (mL/min)		CL_R_ ratio (T2–T3/PP)
		T2–T3	PP	
Creatinine	0.81 (0.78–0.85)	146 (136–157)	117 (111–123)	1.3 (1.2–1.4)
Pyridoxic acid	1.04 (0.85–1.26)	301 (273–331)	226 (208–244)	1.3 (1.2–1.5)
GCDCA‐S	0.58 (0.42–0.8)	21 (17–27)	4 (4–5)	5 (3.8–6.5)
Kynurenic acid	0.64 (0.59–0.7)	125 (101–156)	106 (84–133)	1.2 (0.9–1.5)
p‐Cresol sulfate	0.57 (0.49–0.67)	26 (24–28)	16 (15–18)	1.6 (1.5–1.7)
p‐Cresol glucuronide	0.64 (0.5–0.81)	335 (305–369)	256 (236–277)	1.3 (1.2–1.4)
3‐Indoxyl sulfate	0.6 (0.52–0.69)	98 (87–109)	64 (59–69)	1.5 (1.4–1.7)
Taurine	0.64 (0.58–0.7)	6.8 (5.4–8.5)	7.1 (4.8–10.5)	0.95 (0.7–1.4)
Phenylacetylglutamine	0.68 (0.58–0.79)	NC	NC	1.3 (1.2–1.4)
Kynurenine	0.68 (0.64–0.72)	NC	NC	5.2 (4.5–6.1)
N‐Formylanthranilic acid	0.73 (0.65–0.82)	NC	NC	1.3 (1.1–1.4)

Estimated CL_R_ values were calculated using eqs. 1–3 (in **Supplementary file**). CL_R_, renal clearance; GCDCA‐S, glycochenodeoxycholate‐3‐sulfate; NC, not calculated due to lack of literature data on CL_R_ in healthy adults; PP, postpartum; T2–T3, late second to early third trimester.

### Effect of T2–T3 on plasma concentrations of established and putative biomarkers of renal organic anion transporters (OAT1/3)

**Figure 2:**
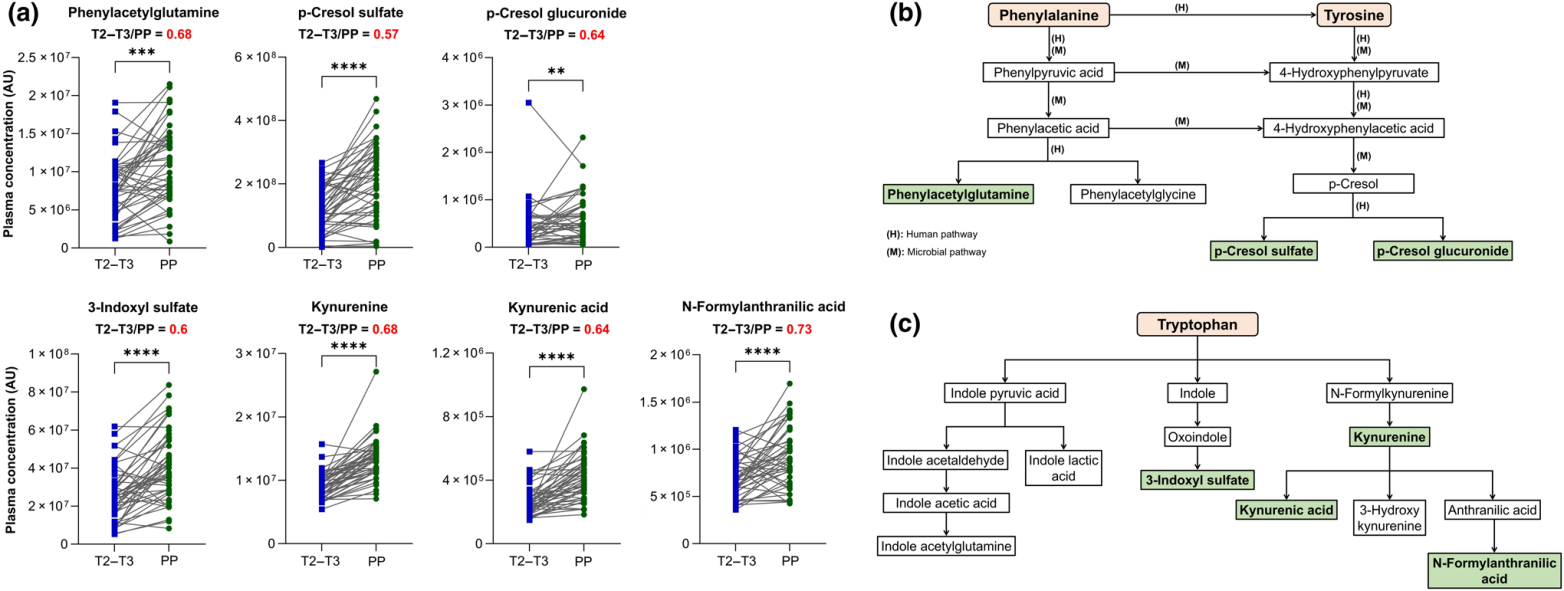
Effect of late second to early third trimester on plasma concentrations of OAT1/3 biomarkers belonging to amino acid pathways. The plasma concentrations of OAT1/3 biomarkers originating from amino acid pathways were 27–43% lower in T2–T3 vs. PP. Line and symbol plots of plasma concentrations of (**a**) OAT1/3 biomarkers belonging to amino acid pathways in pregnant women (*n* = 46) in late second to early third trimester (blue squares, T2–T3) and postpartum (green circles, PP). (**b**) Phenylacetylglutamine, p‐cresol sulfate, and p‐cresol glucuronide are metabolic products of the phenylalanine and tyrosine pathway. **(c)** 3‐indoxyl sulfate, kynurenine, kynurenic acid, and N‐formylanthranilic acid are metabolic products of the tryptophan pathway. Plasma concentrations were compared using the paired *t*‐test. Adjusted *P*‐value < 0.005 (**), < 0.001 (***), and < 0.0001 (****). T2–T3/PP ratios are geometric means (*n* = 46). AU, arbitrary units; PP, postpartum; T2–T3, late second to early third trimester.

**Figure 3:**
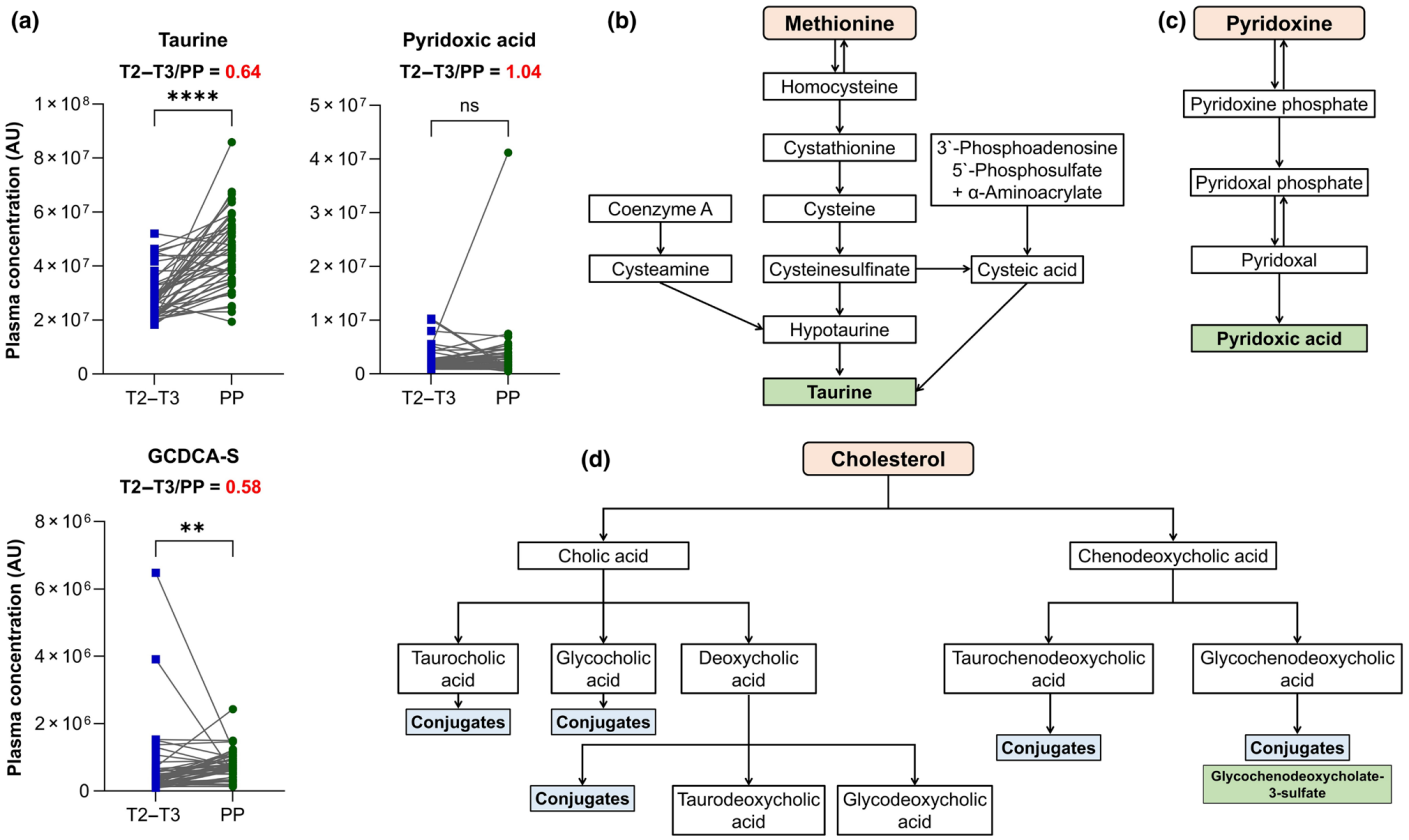
Effect of late second to early third trimester on plasma concentrations of OAT1/3 biomarkers belonging to different biological pathways. The plasma concentrations of taurine and GCDCA‐S were 36% and 42% lower in T2–T3 vs. PP. Non‐significant change in pyridoxic acid plasma concentration is potentially due to high variability in the data. Line and symbol plots of plasma concentrations of (**a**) OAT1/3 biomarkers in pregnant women (*n* = 46) in late second to early third trimester (blue squared, T2–T3) and postpartum (green circles, PP) belonging to (**b**) taurine, (**c**) vitamin B6, and (**d**) bile acid pathways. Pyridoxic acid is the catabolic end product of vitamin B6 pathway, and GCDCA‐S is a conjugate metabolite formed from sequential metabolism of cholesterol. Plasma concentrations were compared using the paired *t*‐test. Adjusted *P*‐value < 0.005 (**) and <0.0001 (****). T2–T3/PP ratios are geometric means (*n* = 46). AU, arbitrary units; GCDCA‐S, glycochenodeoxycholate‐3‐sulfate; ns, non‐significant; PP, postpartum; T2–T3, late second to early third trimester.

Effect of T2–T3 on the plasma concentration of OAT1/3 biomarkers originating from different metabolic pathways was assessed. The average plasma concentration of phenylacetylglutamine, belonging to the phenylalanine pathway, was 32% lower in T2–T3 vs. PP (adjusted *P*‐value < 0.05, **Figure** **2a,b**, **Table** **1**). Plasma concentrations of p‐cresol conjugates (sulfate and glucuronide), catabolic end‐products of tyrosine, were 43% and 36% lower in T2–T3 vs. PP, respectively (adjusted *P*‐value < 0.05, **Figure** **2a,b**, **Table** **1**). The average plasma concentrations of the metabolites of the tryptophan pathway, 3‐indoxyl sulfate, kynurenine, kynurenic acid, and N‐formylanthranilic acid, were 27–40% lower in T2–T3 vs. PP (adjusted *P*‐value < 0.05, **Figure** **2a,c**, **Table** **1**). The average plasma concentrations of GCDCA‐S and taurine were 42% and 36% lower in T2–T3 vs. PP, respectively (adjusted *P*‐value < 0.05, **Figure** **3**, **Table** **1**). The average plasma concentration of pyridoxic acid, a catabolic end‐product of the pyridoxine (Vitamin B6) pathway, was not significantly different between T2–T3 and PP (**Figure** **3**, **Table** **1**). Of the > 50 putative OAT1/3 substrates listed by Granados *et al*.,^20^ a total of 37 metabolites showed significant decrease (> 20%) in plasma concentrations in T2–T3 vs. PP, with data of 32 metabolites shown in **Table** **S4** (the remaining five, 3‐indoxyl sulfate, kynurenine, N‐formylanthranilic acid, p‐cresol glucuronide, and p‐cresol sulfate are shown in **Table** **1**). These metabolites (**Table** **S4**) belong to different metabolic pathways: steroids (androsterone glucuronide), benzoic acid metabolism (4‐ethylcatechol sulfate), chemicals (3‐hydroxy‐2‐methylpyridine sulfate), fatty acid (N‐acetyl‐2‐aminooctanoic acid), food component (dihydrocaffeate sulfate), bile acid metabolism (glycoursodeoxycholic acid), tryptophan metabolism (indoleacetic acid), tyrosine metabolism (4‐hydroxyphenylpyruvic acid), and xanthine metabolism (1‐methyluric acid). Overall, the plasma concentrations of established and putative OAT1/3 biomarkers were significantly lower in T2–T3 vs. PP. Alteration in plasma concentrations of OAT1/3 biomarkers can occur due to changes in either synthesis or elimination and is addressed below.

### Effect of T2–T3 on the amounts of OAT1/3 biomarkers excreted unchanged in the urine

A validated LC‐MS/MS method was used for the quantification of OAT1/3 biomarkers in the urine. The method was accurate (80–120%) and precise (±20%) over a linear range 0.01–1.28 μg/mL for GCDCA‐S, 3‐indoxyl sulfate, p‐cresol glucuronide, p‐cresol sulfate, and phenylacetylglutamine; 0.02–0.64 μg/mL for kynurenic acid; 0.02–1.28 μg/mL for N‐formylanthranilic acid; 0.04–1.28 μg/mL kynurenine and pyridoxic acid; and 0.08 to 1.28 μg/mL for taurine (*r* ^2^ > 0.98). The arbitrary amounts of pyridoxic acid, GCDCA‐S, and kynurenine excreted unchanged in the urine were significantly higher in T2–T3 vs. PP (1.4‐, 2.9‐, and 3.6‐fold, respectively, **Figure** **S2**). The arbitrary amount of taurine excreted unchanged in the urine was 39% lower in T2–T3 vs. PP (*P*‐value < 0.5, **Figure** **S2**). However, the arbitrary amounts of other OAT1/3 biomarkers (kynurenic acid, p‐cresol sulfate, p‐cresol glucuronide, 3‐indoxyl sulfate, phenylacetylglutamine, and N‐formylanthranilic acid) excreted unchanged in the urine were not significantly different between T2–T3 and PP (**Figure** **S2**).

### Changes in CL_R_ of OAT1/3 biomarkers in T2–T3

**Figure 4:**
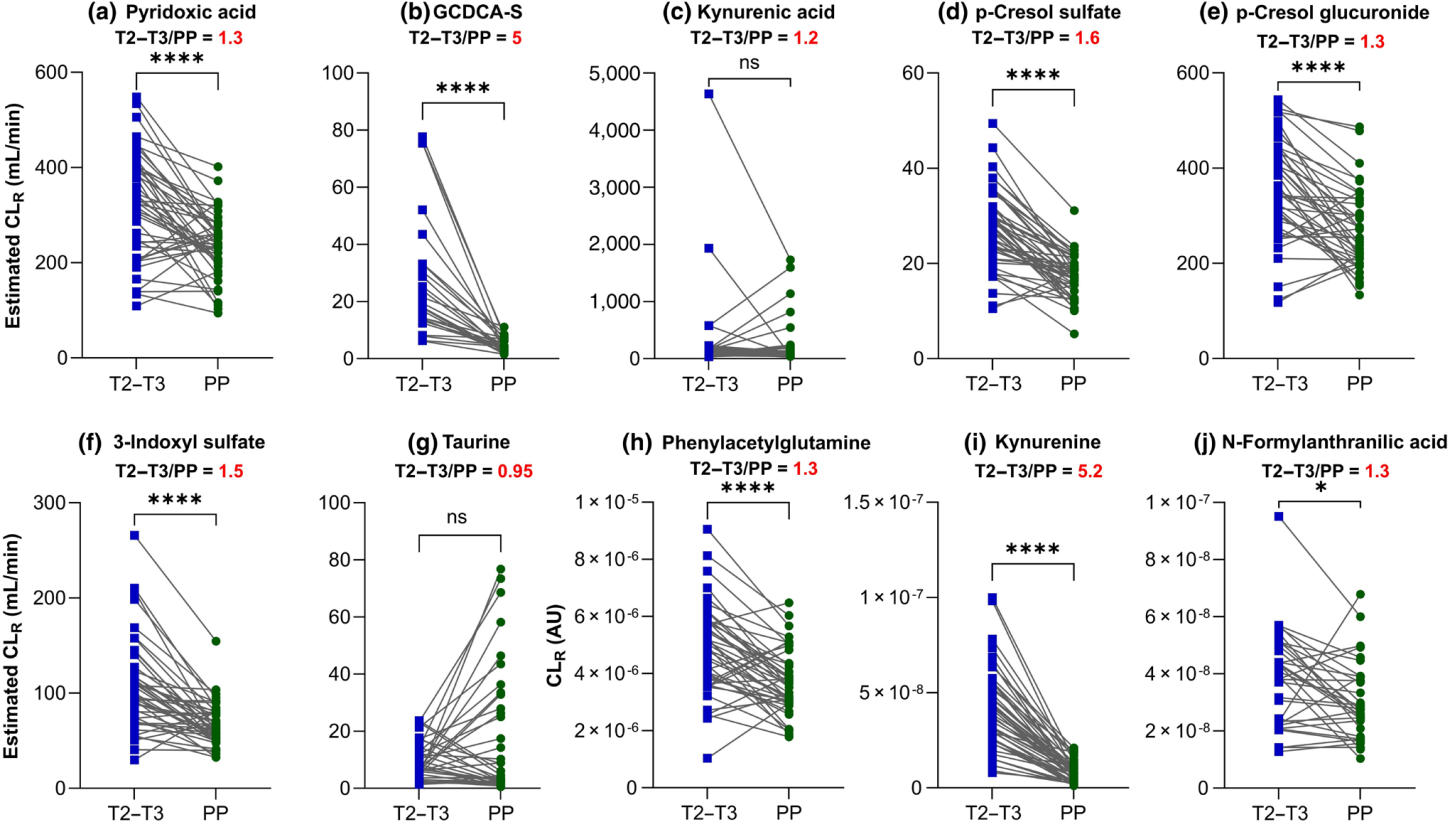
Effect of late second to early third trimester on the renal clearance (CL_R_) of OAT1/3 biomarkers. These data indicate that OAT1/3 activity is induced by ~1.3 to 1.6‐fold in T2–T3 vs. PP. As to why GCDCA‐S and kynurenine CL_R_ values were ~5‐fold in T2–T3 vs. PP needs further investigation. Line and symbol plots of renal clearance (CL_R_) of (**a–j**) OAT1/3 biomarkers in pregnant women (*n* = 46) in late second to early third trimester (blue squares, T2–T3) and postpartum (green circles, PP). CL_R_ values were compared using paired *t*‐test. *P*‐value < 0.05 (*) and < 0.0001 (****). CL_R_ values were estimated using Eqs. 1–3 (in **Supplementary file**). T2–T3/PP ratios are geometric means (*n* = 46). AU, arbitrary units; GCDCA‐S, glycochenodeoxycholate‐3‐sulfate; ns, non‐significant; PP, postpartum; T2–T3, late second to early third trimester.

Since the plasma concentrations of OAT1/3 biomarkers can be altered by not only changes in their elimination but also synthesis, a direct measure of the effect of T2–T3 on their renal excretion was estimated, namely, CL_R_. In T2–T3, the average estimated CL_R_ of pyridoxic acid, an established OAT1/3 biomarker, was 1.3‐fold of that in PP (*P*‐value < 0.05). The average estimated CL_R_ of p‐cresol conjugates in T2–T3 was 1.6‐ and 1.3‐fold of that in PP. Similarly, the average estimated CL_R_ values of phenylacetylglutamine, 3‐indoxyl sulfate, and N‐formylanthranilic acid in T2–T3 were 1.3‐ to 1.5‐fold of PP (*P*‐value < 0.05, **Figure** **4**, **Table** **1**). The average estimated CL_R_ of GCDCA‐S and kynurenine in T2–T3 was ~5‐fold of that in PP (**Figure** **4**, **Table** **1**). The CL_R_ values of kynurenic acid and taurine were not significantly different between T2–T3 and postpartum (**Figure** **4**, **Table** **1**).

### Changes in net secretory clearance (CL_sec_ ) and net unbound CL_sec_ (CL_sec__,u_) of OAT1/3 biomarkers in T2–T3

The CL_R_ of OAT1/3 biomarker is the net outcome of its clearance due to filtration, secretion and reabsorption. Assuming that the reabsorption of the biomarker is negligible and transporter uptake clearance is the rate‐determining step and much less than renal blood flow, the net CL_sec,u_ of the biomarker can be interpreted as a measure of its OAT1/3‐mediated clearance. Therefore, net CL_sec_ and subsequently, net CL_sec,u_ of selected OAT1/3 biomarkers were determined. To do so, the effect of T2–T3 on the plasma protein binding of OAT1/3 biomarkers was determined (*n* = 12). The average f_u,plasma_ values (*n* = 12) of pyridoxic acid, GCDCA‐S, kynurenic acid, p‐cresol sulfate, 3‐indoxyl sulfate, kynurenine, and N‐formylanthranilic acid were not significantly different between T2–T3 and PP (**Table** **S5**, **Figure** **S3**). Phenylacetylglutamine and p‐cresol glucuronide were negligibly bound to plasma proteins (f_u,plasma_ ≈ 1). For taurine, f_u,plasma_ could not be determined due to assay sensitivity.

**Figure 5:**
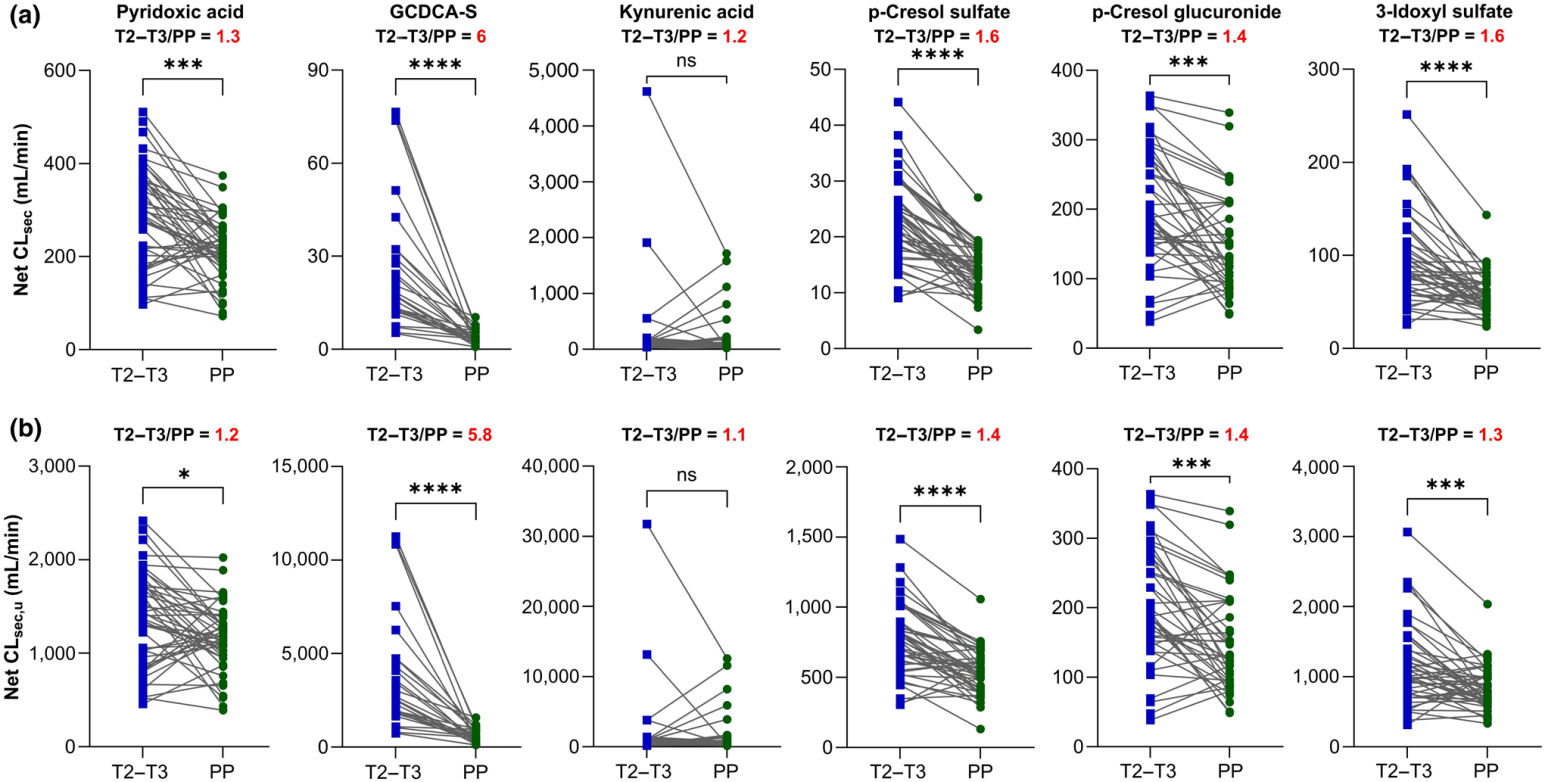
Effect of late second to early third trimester on the net secretory clearance (CL_sec_) and net unbound CL_sec_ (CL_sec,u_) of OAT1/3 biomarkers. These data indicate that OAT1/3 activity is induced by 1.2‐ to 1.4‐fold in T2–T3 vs. PP. As to why net CL_sec_ and net CL_sec,u_ of GCDCA‐S were > 5‐fold higher in T2–T3 vs. PP needs further investigation. Line and symbol plots of (**a**) net secretory clearance (CL_sec_, *n* = 46); and (**b**) net unbound CL_sec_ (CL_sec,u_, *n* = 46) of OAT1/3 biomarkers in pregnant women, in late second to early third trimester (blue square, T2–T3) and postpartum (green circles, PP). Net CL_sec_ and net CL_sec,u_ values were compared using the paired *t*‐test. *P*‐value < 0.05 (*), < 0.001 (***), and < 0.0001 (****). Net CL_sec_ values were calculated using Eq. 5 (in **Supplementary file**). For calculating net CL_sec,u_ (*n* = 46), average f_u,plasma_ values in *n* = 12 subjects were used. T2–T3/PP ratios are geometric means (*n* = 46). GCDCA‐S, glycochenodeoxycholate‐3‐sulfate; ns, non‐significant; PP, postpartum; T2–T3, late second to early third trimester.

The net CL_sec_ values were determined in 46 study participants, using the average f_u,plasma_ values of the biomarkers (to determine CL_filtration_). The net CL_sec_ values of pyridoxic acid, p‐cresol sulfate, p‐cresol glucuronide, and 3‐indoxyl sulfate in T2–T3 were 1.3‐ to 1.6‐fold of PP (*P*‐value < 0.05, **Figure** **5a**). In contrast, the net CL_sec_ of GCDCA‐S in T2–T3 was 6‐fold of PP (*P*‐value < 0.05, **Figure** **5a**). Kynurenic acid net CL_sec_ was not significantly different between T2–T3 and PP. The net CL_sec_ of phenylacetylglutamine, kynurenine, and N‐formylanthranilic acid could not be calculated due to lack of their literature reported CL_R_ values in healthy adults. For taurine, although CL_R_ values are available,^17^, ^26^ its CL_sec_ values could not be determined as its fraction reabsorbed is not known. The net CL_sec_ values determined in 12 study participants (**Figure** **S4a**), using individual f_u,plasma_ values of the biomarkers, were similar to those described above (*n* = 46).

Net CL_sec,u_ in the 46 study participants was estimated using the average f_u,plasma_ in the 12 participants. The net CL_sec,u_ values of pyridoxic acid, p‐cresol sulfate, p‐cresol glucuronide, and 3‐indoxyl sulfate were significantly higher in T2–T3 vs. PP (1.2‐ to 1.4‐fold, *P*‐value < 0.05, **Figure** **5b**, **Table** **2**). In the case of GCDCA‐S, net CL_sec,u_ was significantly greater in T2–T3 vs. PP (5.8‐fold, *P*‐value < 0.05, **Figure** **5b**, **Table** **2**). As for the net CL_sec,u_ calculated in 12 participants using individual f_u,plasma_ values, they were not significantly different between T2–T3 vs. PP for pyridoxic acid and p‐cresol sulfate (**Figure** **S4b**, **Table** **S6**). This is potentially due to small sample size. For the other biomarkers, net CL_sec,u_ values determined in 12 study participants were similar to those described above (*n* = 46). Complete data of amount excreted unchanged in urine, CL_R_, net CL_sec_, and net CL_sec,u_ are available on Open Science Framework (project URL is https://osf.io/a4329 with the registered DOI: https://doi.org/10.17605/OSF.IO/3PFQE).^27^

**Table 2:** Geometric means (90% confidence interval) of net unbound secretory clearance (CL_sec,u_) of established and putative OAT1/3 biomarkers in pregnant women (*n* = 46) during late second to early third trimester (T2–T3) and postpartum (PP)

Metabolite	Estimated net CL_sec,u_ (mL/min)		Net CL_sec,u_ ratio (T2–T3/PP)
	T2–T3	PP	
Pyridoxic acid	1,270 (1,144–1,409)	1,096 (1,003–1,197)	1.2 (1.04–1.3)
GCDCA‐S	2,893 (2,241–3,735)	497 (391–634)	5.8 (4.3–7.9)
Kynurenic acid	675 (529–863)	615 (474–798)	1.1 (0.9–1.4)
p‐Cresol sulfate	724 (664–789)	518 (476–564)	1.4 (1.3–1.5)
p‐Cresol glucuronide	182 (158–209)	133 (118–151)	1.4 (1.2–1.6)
3‐Indoxyl sulfate	1,033 (917–1,163)	782 (717–853)	1.3 (1.2–1.5)

Net CL_sec,u_ values were calculated using Eqs. 4–5 (in **Supplementary file**). GCDCA‐S, glycochenodeoxycholate‐3‐sulfate; Net CL_sec,u_, unbound renal secretory clearance; PP, postpartum; T2–T3, late second to early third trimester.

### Correlations of net CL_sec_ _,u_ of OAT1/3 biomarkers

In the 46 study participants, the correlation of net CL_sec,u_ of pyridoxic acid with p‐cresol sulfate, p‐cresol glucuronide, and 3‐indoxyl sulfate in T2–T3 was higher than in PP (coefficient of determination, *R* ^2^ > 0.5, **Figure** **S5**). Similarly, net CL_sec,u_ of p‐cresol sulfate, p‐cresol glucuronide, and 3‐indoxyl sulfate were more strongly correlated in T2–T3 than PP. The net CL_sec,u_ of GCDCA‐S did not correlate (*R* ^2^ ≤ 0.1) with the net CL_sec,u_ of other biomarkers in either T2–T3 or PP (**Figure** **S5**).

## DISCUSSION

In this proof‐of concept study, we utilized a biomarker approach to quantify the effect of T2–T3 of pregnancy on renal OAT1/3 activity and compared this biomarker approach to data published in the literature where changes in clearance (CL) of drugs during T2 or T3 were used to report renal OAT1/3 activity.^9^, ^14^ There are several unique aspects of our study or study design that should be noted. First, to our knowledge, this is a first study that utilized multiple biomarkers (from different metabolic pathways) to interrogate pregnancy‐related changes in OAT1/3 activity. The use of multiple OAT1/3 biomarkers, originating from different metabolic pathways (e.g. amino acid pathways, bile acid pathway, steroids, xanthine, vitamins) enhanced our confidence in determining if OAT1/3 activity is induced during T2–T3. Second, this *in vivo* study was carried out in a large cohort (*n* = 46) of healthy pregnancies using paired samples obtained in T2–T3 and PP allowing increased power to quantify changes in OAT1/3 activity. Third, our study was carried out in healthy women with uncomplicated pregnancy which minimized the confounding effect of pregnancy‐related complications.

The steady‐state plasma concentration of a biomarker is dependent on its rate of synthesis and rate of elimination. Thus, any change in this steady‐state plasma concentration cannot be interpreted as a change only in the CL (e.g., CL_R_) of the biomarker. Therefore, we first focused on T2–T3‐induced changes in the CL_R_ of the OAT1/3 biomarkers. Even CL_R_ is not a direct reporter of renal OAT1/3 activity because it can be influenced by changes in plasma protein binding of the biomarker, glomerular filtration and renal blood flow, which can all change during pregnancy (see below for further discussion). Because net CL_sec_ and net CL_sec,u_ are more proximate measures of OAT1/3 activity, next, we focused on these parameters. The assertion that net CL_sec_ and net CL_sec,u_ report renal OAT1/3 activity is predicated on the following assumptions: (i) OAT1/3‐mediated uptake is the rate‐determining step in biomarker net CL_sec_ and is much less than renal blood flow; and (ii) pregnancy does not result in any changes in the reabsorption CL of the biomarker. These are reasonable assumptions as the estimated net CL_sec_ values of OAT1/3 biomarkers are much less than the renal blood flow (~1,200 mL/min) (**Figure** **4**, and **Table** **1**). Furthermore, most of the studied biomarkers are ionized at urine pH (pKa ≈ −2.0 to 3.9), resulting in little, if any, passive reabsorption of the biomarker across the apical membrane of the proximal tubular epithelial cells. However, we cannot discount the possibility of active reabsorption *via* OAT4 (or other uptake transporters), localized on the apical membrane of proximal tubular epithelial cells. These biomarkers are also mostly ionized at physiological pH resulting in minimal passive diffusion unbound CL (CL_u_) from the intracellular compartment of the proximal tubular epithelial cells to blood across the basal membrane. Provided this passive diffusion CL_u_ is much lower than the apical efflux CL_u_ from the proximal tubular epithelia cells, one can assume that OAT1/3‐mediated uptake CL_u_ of the biomarker is the rate‐determining step in its CL_sec,u_.^28^

The CL_R_ ratios (T2–T3/PP) of the biomarkers, that were significantly altered in T2–T3, fell into two distinct groups. In group 1, the CL_R_ ratios of biomarkers, consisting of pyridoxic acid, phenylacetylglutamine, p‐cresol sulfate, p‐cresol glucuronide, 3‐indoxyl sulfate, and N‐formylanthranilic acid, were ~1.5 (*P*‐value < 0.05). In contrast, the CL_R_ ratios in group 2, consisting of GCDCA‐S and kynurenine, were ~ 5 (*P*‐value < 0.05). These findings indicate enhanced renal OAT1/3 activity in T2–T3. Possible reasons why the biomarkers may have been segregated into two distinct groups are discussed below. Overall, these data are by and large consistent with the significant decrease in biomarker steady‐state plasma concentration except for the CL_R_ ratios of kynurenic acid and taurine which were not significantly changed in T2–T3. Significant decrease in plasma concentrations of kynurenic acid (a putative OAT1/3 biomarker^19^), in T2–T3, without a corresponding increase in CL_R_ hints at the involvement of pathways other than OAT1/3 in the renal CL_R_ of kynurenic acid. Similarly, CL_R_ of taurine was not altered in T2–T3, but its steady‐state plasma concentration was significantly decreased in the plasma (**Figures** **2**, **3**, **4**, **Table** **1**). Taurine CL_R_ is plausibly confounded by reabsorption *via* a sodium‐dependent transporter (TauT).^17^, ^29^ In contrast, pyridoxic acid steady‐state plasma concentration was not altered in T2–T3, while its CL_R_ was induced (**Figures** **3** **and** **4**, **Table** **1**). Non‐significant change in pyridoxic acid plasma concentration is potentially due to high variability which can arise from differential intake of the precursor, that is, vitamin B6 during T2–T3 vs. PP.^6^, ^16^ These discrepancies between CL_R_ and steady‐state plasma concentrations reinforce the notion that interpretation of the steady‐state plasma concentrations of biomarkers can be confounded by factors (e.g., synthesis and diet) other than CL_R_.

As indicated earlier, biomarker net CL_sec_ and net CL_sec,u_ values are better predictors of OAT1/3 activity than CL_R_. The net CL_sec_ of group 1 biomarkers (pyridoxic acid, p‐cresol sulfate, p‐cresol glucuronide, and 3‐indoxyl sulfate) were 1.3‐ to 1.6‐fold higher in T2–T3 vs. PP. Furthermore, pyridoxic acid, p‐cresol sulfate, p‐cresol glucuronide, and 3‐indoxyl sulfate net CL_sec,u_ values (*n* = 46) in T2–T3 were 1.2‐ to 1.4‐fold of postpartum. This is consistent with our earlier conclusion (based on CL_R_) on the induction of renal OAT1/3‐activity in T2–T3. Overall, these findings are consistent with the literature reported data of OAT1/3 substrate drugs (amoxicillin, cefazolin, oseltamivir, pravastatin, and tenofovir), wherein CL_R_ (as well as net CL_sec_ and net CL_sec,u_) in T2–T3 are ~1.3 to 1.8‐fold of PP values (**Table** **S7**).^7^, ^9^, ^12^, ^13^, ^14^, ^15^

For the group 2 biomarker, GCDCA‐S, net CL_sec_ and net CL_sec,u_ values in T2–T3 were 5.8‐ to 8.2‐fold of PP. We postulate various scenarios which could explain the differences in CL_R_, net CL_sec_ or net CL_sec,u_ between group 1 and 2 biomarkers. First, GCDCA‐S and kynurenine may be selective substrates of another uptake transporter localized on the basal membrane of proximal tubule cells. Pregnancy‐mediated increase in the activity of this transporter might contribute to the overall increase in the CL_R_ of these metabolites. Second, group 1 biomarkers underestimate the true OAT1/3 activity and therefore their induction in T2–T3. This is possible if OAT‐mediated secretion is not the sole rate‐determining step in the net CL_sec_ of these biomarkers. This could occur due to the presence of a basal efflux transporter in proximal tubule cells, such that basal efflux clearance for these biomarkers (except GCDCA‐S and kynurenine) is not significantly lower than the apical efflux clearance. Alternatively, the fraction transported by group 1 vs. group 2 biomarkers *via* OAT1 or OAT3 may differ. These hypotheses are supported by the lack of correlation of GCDCA‐S net CL_sec,u_ with net CL_sec,u_ of group 1 biomarkers (**Figure** **S5**). In contrast, the net CL_sec,u_ values of group 1 biomarkers were strongly correlated in both T2–T3 and PP, with stronger correlations in T2–T3. This suggests that: (i) in T2–T3, the majority of variability in net CL_sec,u_ values of group 1 biomarkers can be explained by induction of OAT1/3 activity; and (ii) net CL_sec,u_ values of group 1 biomarkers are reporting altered activity of a common elimination pathway. Overall, net CL_sec,u_ of OAT1/3 biomarkers is significantly higher in late second to early third trimester, compared to postpartum. While the mechanism of this alteration is not well studied, we postulate that pregnancy‐related hormones can induce the expression of OAT1/3 in human proximal tubule epithelial cells. This hypothesis is supported by an increase in the mRNA expression and activity of OAT2 in human hepatocytes and HepaRG cells exposed to a cocktail of pregnancy‐related hormones.^30^, ^31^

As was the case with CL_R_, the net CL_sec_ and net CL_sec,u_ of kynurenic acid were not significantly different between T2–T3 and PP. Net CL_sec_ and net CL_sec,u_ values could not be calculated for phenylacetylglutamine, kynurenine, and N‐formylanthranilic acid due to the absence of literature reported CL_R_ values in healthy adults. While CL_R_ values were available for taurine, its net CL_sec_ and net CL_sec,u_ values were not calculated due to the lack of data on the fraction of taurine reabsorbed in T2–T3 and non‐pregnant individuals.

Our study has limitations that could be addressed by future work. Ideally, the intrinsic unbound net CL_sec_ of OAT1/3 biomarkers (i.e., considering pregnancy‐induced changes in renal blood flow) would be a better reporter of changes in OAT1/3 activity. The lack of blood to plasma ratio data for these biomarkers in T2–T3 and postpartum precluded the estimation of these intrinsic unbound net CL_sec_ values. Creatinine was used as a CL_filtration_ marker. However, creatinine CL_R_ can be corrupted by varying degrees of OCT2‐mediated active renal secretion. In future, cystatin‐C, which is not transported, could be used as a GFR marker.^32^, ^33^ OAT1 and OAT3 share high sequence similarity (49.6%), have overlapping substrate specificity, and their expression is transcriptionally co‐regulated in healthy individuals.^34^, ^35^ However, the regulatory effect of pregnancy on the expression and function of OAT1 and OAT3 has not been investigated and could be distinct from non‐pregnant individuals. The recent discovery of selective OAT1 and OAT3 biomarkers^22^ will allow future studies to delineate the differential impact of pregnancy on OAT1 vs. OAT3 activity. These selective biomarkers could not be quantified here as we utilized plasma metabolomics data retrospectively generated by Metabolon Inc. and their metabolite panel does not contain these selective biomarkers. Since most OAT1/3 biomarkers are acidic and ionized at urine pH, passive reabsorption should be minimal. Therefore, any increase in urine pH during pregnancy^36^, ^37^ will have minimal effect on the ionization and passive reabsorption of these acidic biomarkers. Furthermore, CL_R_ values were determined using the midpoint method which assumes minimal intraindividual changes in plasma concentrations during the urine collection time interval, an assumption not made by the more robust AUC method.^38^ The latter was not possible as this was an opportunistic study utilizing existing midpoint plasma samples. Furthermore, the differential effect of single‐dose dextromethorphan administration on renal transporters between T2–T3 and PP could confound our findings. However, no data exist in the literature that dextromethorphan or its metabolites can inhibit renal OAT1/3 activity. Since this was an opportunistic study, dietary restrictions could not be incorporated in the study design. The precursors of various OAT1/3 biomarkers (pyridoxic acid, kynurenic acid, p‐cresol conjugates, etc.) are derived from diet (food and supplements), which can potentially alter biomarker plasma concentrations. Here, we quantified the net CL_sec_ and net CL_sec,u_ values of the biomarkers. Theoretically, under linear pharmacokinetics, these values are independent of biomarker plasma concentrations and dependent only on renal OAT1/3 activity.

This is a proof‐of‐concept study utilizing multiple renal OAT1/3 biomarkers originating from different biological pathways to evaluate T2–T3‐mediated modulation of renal OAT1/3 activity. Net CL_sec,u_ of all the studied biomarkers (except GCDCA‐S) in T2–T3 was 1.2‐ to 1.4‐fold of PP. These changes are similar to the published OAT1/3 probe substrate data.^7^, ^9^, ^12^, ^13^, ^14^, ^15^ As to why GDCA‐S has an even larger change in OAT1/3 activity, as determined by net CL_sec_ and net CL_sec,u_, in T2–T3 needs further investigation. These data support the premise that the biomarker approach can be used as a non‐invasive tool to study the effect of pregnancy on the activity of drug metabolizing enzymes and transporters (DMETs). We intend to utilize group 1 and group 2 biomarkers to evaluate alterations in the activity of renal OAT1/3 across all three trimesters. After additional investigation of the reason for differences in net CL_sec_ and net CL_sec,u_ values between groups 1 and 2, a panel of biomarkers will be chosen to inform dosing of OAT1/3 substrate drugs during pregnancy.

In the future, this work will be extended to evaluate longitudinal changes (across all three trimesters) in the activity of renal OAT1/3 and other renal and hepatic DMET proteins (including organic cation transporters 1 and 2, organic anion transporting polypeptide 1B1 and 1B3, CYP1A2, CYP3A4, CYP2D6, etc.) during pregnancy compared to postpartum by untargeted metabolomic analysis of plasma and urine samples. Our work represents the first systematic evaluation of OAT1/3 biomarkers to predict pregnancy‐induced changes in transport activity. With CL_R_, net CL_sec_, and net CL_sec,u_ changes comparable to probe substrate data, these biomarkers can be utilized either directly or can be incorporated into physiologically based pharmacokinetic models to inform precision dosing of renal OAT1/3 substrates (e.g., antivirals, antibiotics) administered during T2–T3.

## FUNDING

The work was supported by the National Institute of General Medical Sciences (NIGMS) [Grant R01GM124264], the Eunice Kennedy Shriver National Institute of Child Health and Human Development (NICHD) [Grant R01HD112282], and the Gates Foundation [Grant INV‐006678]. NI is supported in part by the Milo Gibaldi Endowed Chair of Pharmaceutics awarded to the Department of Pharmaceutics, University of Washington.

## CONFLICTS OF INTEREST

The authors declared no competing interests for this work.

## DISCLOSURE

As an Editor‐in‐Training for *Clinical Pharmacology & Therapeutics*, Aarzoo Thakur was not involved in the review or decision‐making for this paper.

## AUTHOR CONTRIBUTIONS

A.T., J.D.U., E.E.F., N.I., and M.F.H. wrote the manuscript. A.T., J.D.U., and M.F.H. designed the research. A.T., J.D.U., and M.F.H. performed the research. A.T. and J.D.U. analyzed the data. E.E.F., N.I., and M.F.H contributed new reagents/analytical tools.

## Supporting information


Data S1
