# Supplementary material for: Effect of Late Second to Early Third Trimester of Pregnancy on the Activity of Renal Organic Anion Transporters (OAT1 and OAT3): A Biomarker Study
Source: Clin Pharmacol Ther. 2026 May 11;120(2):465–74. doi: 10.1002/cpt.70321 (PMC13339035; doi:10.1002/cpt.70321)
Supplement: Supplementary file 1 — Data S1 [file CPT-120-465-s001.docx]

**SUPPLEMENTARY INFORMATION**

**Effect of late second to early third trimester of pregnancy on the activity of renal organic anion transporters (OAT1 and OAT3): A biomarker study**

Aarzoo Thakur^1^, Jashvant D. Unadkat^1^*, Emily E. Fay^2^, Nina Isoherranen^1^, and Mary F. Hebert^2,3^*

^1^Department of Pharmaceutics, School of Pharmacy, University of Washington, Seattle, WA, United States

^2^Department of Obstetrics and Gynecology, School of Medicine, University of Washington, Seattle, WA, United States

^3^Department of Pharmacy, School of Pharmacy, University of Washington, Seattle, WA, United States

***CORRESPONDING AUTHORS**

Mary F. Hebert ([mhebert@uw.edu](mailto:mhebert@uw.edu))

Department of Pharmacy

School of Pharmacy

University of Washington

Seattle, WA

and

Jashvant D. Unadkat ([jash@uw.edu](mailto:jash@uw.edu))

Department of Pharmaceutics

School of Pharmacy

University of Washington

Seattle, WA

**SUPPLEMENTARY METHODS**

**Materials**

Cimetidine, N-formylanthranilic acid, human serum albumin (HSA), 3-indoxyl sulfate potassium salt, L-kynurenine, kynurenic acid, nifedipine, phenylacetyl-L-glutamine, pyridoxic acid, sodium chloride, taurine, and trimethoprim were purchased from Sigma-Aldrich (St. Louis, MO). Acetic acid, formic acid, LCMS-grade acetonitrile, LCMS-grade water, sodium phosphate dibasic anhydrous, and sodium phosphate monobasic monohydrate were procured from Fisher Chemical (Fair Lawn, NJ). Creatinine-d3, p-cresol glucuronide, and p-cresol sulfate potassium salt were obtained from Cayman Chemical (Ann Arbor, MI). Glycochenodeoxycholate-3-sulfate (GCDCA-S) was procured from Toronto Research Chemicals (Toronto, Canada). Creatinine, and olmesartan were purchased from Acros Organics (Fair Lawn, NJ) and Tocris Bioscience (Minneapolis, MI), respectively. Rapid Equilibrium Dialysis (RED) kit was obtained from Thermo Fisher Scientific (Rockford, IL).

**Development and validation of LC-MS/MS-based metabolomics method for analysis of urine samples**

The developed LC-MS/MS method was validated for linearity, accuracy, precision, and autosampler stability using metabolite standards prepared with water as a matrix. The stock solutions of following compounds were prepared: creatinine (5 mg/mL), p-cresol sulfate (5 mg/mL), and taurine (5 mg/mL) in water; and N-formylanthranilic acid (4.3 mg/mL), GCDCA-S (3.6 mg/mL), 3-indoxyl sulfate (5 mg/mL), kynurenine (5 mg/mL), kynurenic acid (5 mg/mL), p-cresol glucuronide (5 mg/mL), phenylacetylglutamine (5 mg/mL), and pyridoxic acid (2 mg/mL) in dimethylsulfoxide. Calibration solutions for linearity assay ranged from 0.01 to 1.28 µg/mL for GCDCA-S, 3-indoxyl sulfate, p-cresol glucuronide, p-cresol sulfate, and phenylacetylglutamine; 0.02 to 0.64 µg/mL for kynurenic acid; 0.02 to 1.28 µg/mL for N-formylanthranilic acid; 0.04 to 1.28 µg/mL kynurenine and pyridoxic acid; 0.08 to 1.28 µg/mL for taurine; and 0.16 to 1.28 µg/mL for creatinine. Quality control (QC) samples consisted of low QC (at lowest concentration of calibration curve), mid QC (at 160 ng/mL), and high QC (at 640 ng/mL).

The processed urine samples (including for method validation) were analyzed using Waters ACQUITY ultra-performance liquid chromatography (UPLC) coupled with Xevo TQ-S instrument containing electrospray ionization source. The chromatographic separation was achieved with ACQUITY UPLC® HSS T3 (1.8 µm; 100x2.1 mm) column set at 40 °C. The mobile phase flow rate was 300 µL/min. The samples were analyzed in both positive and negative ionization modes. For positive mode, 0.1% formic acid in water and 0.1% formic acid in acetonitrile were used as mobile phase A and B, respectively. For the negative mode, mobile phases A and B consisted of 0.1% acetic acid in water and 0.1% acetic acid in acetonitrile, respectively. Details about LC gradient and MS parameters are provided in Table S1. The optimized multiple reaction monitoring parameters for metabolites are listed in Table S2.

**Preparation of urine samples**

Fifty µL of urine sample was protein precipitated by adding 50 µL of ice-cold acetonitrile containing internal standards (cimetidine: 25 ng/mL; creatinine-d3: 500 ng/mL; nifedipine: 50 ng/mL; olmesartan: 100 ng/mL; and trimethoprim: 25 ng/mL), vortexed, and centrifuged at 16,000 xg and 4 °C for 10 min. Then, 80 µL of supernatant was diluted with 320 µL of 10:90 acetonitrile: water (v/v) containing 0.1% formic acid, vortexed, and centrifuged at 16,000 xg and 4 °C for 10 min. The supernatant was transferred into LC-MS vial for analysis.

**f_u,plasma_ protocol**

Out of the 46 study participants, the fraction unbound in plasma (f_u,plasma_) of the biomarkers in the late second to early third trimester (T2-T3) and postpartum (PP) plasma samples of 12 randomly chosen participants were quantified in duplicate using the Thermo Scientific™ RED plate. The plasma samples were spiked with 10x concentrations of the metabolites to get a final concentration of 7.5 µM for pyridoxic acid, 50 µM for GCDCA-S, 10 µM for kynurenic acid, 20 µM for p-cresol sulfate, 5 µM for p-cresol glucuronide, 15 µM for 3-indoxyl sulfate, 7.5 µM for taurine, 5 µM for kynurenine, and 10 µM for N-formylanthranilic acid. Final metabolite concentrations were chosen based on the sensitivity of the LC-MS/MS assay to accurately quantitate both buffer and plasma chamber concentrations. Dialysis buffer consists of phosphate-buffered saline containing 100 mM sodium phosphate and 150 mM sodium chloride, with pH adjusted to 7.4. Fifty µL of plasma was aliquoted into the plasma chamber and 300 µL of dialysis buffer into the buffer chamber, followed by incubation at 37 °C and 250 rpm for 4 h. Thereafter, 30 µL of sample was aliquoted from the plasma and buffer chambers. To the plasma and buffer samples, 30 µL of dialysis buffer and 1% HSA were added, respectively, followed by protein precipitation with 720 µL and 180 µL of acetonitrile (containing internal standards), respectively. The samples were vortexed, and centrifuged at 16,000 xg and 4 °C for 10 min. The supernatant was dried at 45 °C for 2 h, and reconstituted with 240 µL and 60 µL of 10:90 acetonitrile: water (v/v) containing 0.1% formic acid for plasma and buffer samples, respectively. The reconstituted samples were vortexed, centrifuged at 16,000 xg and 4 °C for 10 min and transferred into LC-MS vial for analysis.

**Data and pharmacokinetic analysis**

LC-MS/MS data obtained from LC-MS analysis of urine and fraction unbound samples were analyzed using Skyline 25.1. The biomarker average product ion peak area was divided by average product ion peak area of the internal standard to get peak area ratio. Based on the relative retention time and ionization mode, various compounds were used as internal standards for different biomarkers (creatinine-d3 for creatinine; cimetidine for pyridoxic acid and kynurenine; trimethoprim for kynurenic acid and phenylacetylglutamine; nifedipine for GCDCA-S; olmesartan for p-cresol sulfate, p-cresol glucuronide, 3-indoxyl sulfate, taurine, and N-formylanthranilic acid). Urine concentrations (in arbitrary units) were converted to urine amounts (in arbitrary units) using the urine volume (in mL). Rate of excretion and CL_R_ (arbitrary units) were calculated using equations 1 and 2.

$$Rate of excretion \left( arbitrary units \right)=\frac{Amount excrete unchanged in urine (arbitrary units)}{Time (in min)} (1)$$

$$\mathrm{CL}_{R}\left( arbitrary units \right)=\frac{Rate of excretion over 4 hr (arbitrary units)}{Midpoint plasma concentration (2 h, arbitrary units)} (2)$$

Since the Metabolon generated plasma data were in arbitrary units, the apparent CL_R_ for each individual (in T2-T3 and PP) was estimated (equation 3) by assuming that the PP biomarker CL_R_ (in arbitrary units), calculated as arithmetic mean across all individuals, is reflective of the literature reported CL_R_ (where available) of the biomarker in healthy adult population.

$${Estimated CL}_{R}\left( mL/min \right)=\frac{\mathrm{Individual}\mathrm{CL}_{R}\left( arbitrary units \right)\times Literature reported \mathrm{CL}_{R} values (mL/min)}{Average PP \mathrm{CL}_{R} (arbitrary units)} (3)$$

Where multiple CL_R_ values were available in the literature (creatinine, pyridoxic acid, kynurenic acid, taurine, and GCDCA-S), weighted average CL_R_ values were used (Table S3). Fraction unbound in plasma was calculated using equation 4 (n=12), followed by calculation of the estimated net CL_sec_ (equation 5). Passive reabsorption was assumed as negligible as the studied biomarkers are ionized at urine pH (pK_a_ ≈ −2.0 to 3.9). Creatinine CL_R_ was used as a marker of CL_filtration_.

$$f_{u,plasma}=\frac{Biomarker concentration in the buffer chamber}{Biomarker concentration in the plasma chamber} (4)$$

$$Estimated net \mathrm{CL}_{\sec} (mL/min) =Estimated \mathrm{CL}_{R}-\left( f_{u,plasma}\times Creatinine \mathrm{CL}_{R} \right) (5)$$

Thereafter, net CL_sec,u_ was calculated as a ratio of net CL_sec_ and f_u,plasma_. Using the average f_u,plasma_ values from n=12, net CL_sec_ and net CL_sec,u_ values were calculated for all 46 study participants. Then, CL_R_, net CL_sec_ and net CL_sec,u_ ratios were calculated using equation 6.

$$\mathrm{CL}_{i} ratio=\frac{\mathrm{Estimated}\mathrm{CL}_{i} in T2-T3}{\mathrm{Estimated}\mathrm{CL}_{i} in PP} (6)$$

where, CL*_i_* represents CL_R_, net CL_sec_ or net CL_sec,u_.

**SUPPLEMENTARY TABLES**

**Table S1. (a) LC gradient and (b) MS tune parameters used in LC-MS/MS analysis.**

**(a)**

| **Positive mode LC time (min)** | **Negative mode LC time (min)** | **%B** |
| --- | --- | --- |
| 0-2 | 0-1 | 5 |
| 2-5 | 1-5 | 55 |
| 5-8.4 | 5-8.4 | 68 |
| 8.4-9.4 | 8.4-9.4 | 90 |
| 9.4-12 | 9.4-12 | 90 |
| 12-12.5 | 12-12.5 | 5 |
| 12.5-15 | 12.5-15 | 5 |

**(b)**

| **Parameter** | **Positive ionization mode** | **Negative ionization mode** |
| --- | --- | --- |
| Capillary voltage (kV) | 3 | 2.5 |
| Desolvation temperature (^o^C) | 350 | 350 |
| Desolvation gas flow (L/h) | 800 | 1000 |
| Cone gas flow (L/h) | 150 | 150 |
| Nebuliser (bar) | 6 | 6 |

**Table S2. Optimized multiple reaction monitoring parameters of metabolites.**

| **#** | **Compound** | **Polarity** | **Precursor ion (m/z)** | **Product ion (m/z)** | **Cone voltage (V)** | **Collision energy (eV)** |
| --- | --- | --- | --- | --- | --- | --- |
| 1 | Creatinine | Positive | 114.0319 | 85.8749 | 25 | 10 |
| 2 | Creatinine-d3 | Positive | 117.0043 | 88.8572 | 28 | 8 |
| 3 | Pyridoxic acid | Positive | 183.9681 | 64.7926 | 22 | 28 |
|  |  |  | 183.9681 | 147.6993 | 22 | 18 |
| 4 | Kynurenic acid | Positive | 190.0319 | 88.7898 | 40 | 33 |
|  |  |  | 190.0319 | 115.7786 | 40 | 26 |
|  |  |  | 190.0319 | 143.6797 | 40 | 18 |
| 5 | Kynurenine | Positive | 209.0319 | 93.8017 | 30 | 8 |
|  |  |  | 209.0319 | 145.7665 | 30 | 18 |
| 6 | Cimetidine | Positive | 253.0957 | 94.7707 | 40 | 20 |
|  |  |  | 253.0957 | 98.8024 | 40 | 22 |
|  |  |  | 253.0957 | 116.7501 | 40 | 14 |
|  |  |  | 253.0957 | 158.7562 | 40 | 12 |
| 7 | Phenylacetylglutamine | Positive | 265.0319 | 83.7767 | 28 | 26 |
|  |  |  | 265.0319 | 90.797 | 28 | 28 |
|  |  |  | 265.0319 | 129.6803 | 28 | 12 |
|  |  |  | 265.0319 | 135.7904 | 28 | 12 |
| 8 | Trimethoprim | Positive | 291.0319 | 109.8459 | 14 | 34 |
|  |  |  | 291.0319 | 122.7843 | 14 | 22 |
|  |  |  | 291.0319 | 229.6728 | 14 | 22 |
|  |  |  | 291.0319 | 260.6193 | 14 | 26 |
| 9 | Nifedipine | Negative | 345.0319 | 91.7687 | 26 | 16 |
|  |  |  | 345.0319 | 121.7371 | 26 | 12 |
|  |  |  | 345.0319 | 221.6574 | 26 | 10 |
|  |  |  | 345.0319 | 312.5412 | 26 | 8 |
| 10 | Olmesartan | Negative | 445.0957 | 108.8034 | 56 | 38 |
|  |  |  | 445.0957 | 148.7863 | 56 | 36 |
|  |  |  | 445.0957 | 166.7299 | 56 | 24 |
|  |  |  | 445.0957 | 372.5575 | 56 | 20 |
| 11 | GCDCA-S | Negative | 528.0957 | 73.8253 | 66 | 42 |
|  |  |  | 528.0957 | 96.7089 | 66 | 58 |
|  |  |  | 528.0957 | 385.6753 | 66 | 46 |
|  |  |  | 528.0957 | 447.5649 | 66 | 32 |
| 12 | Taurine | Negative | 123.9681 | 64.7292 | 56 | 12 |
|  |  |  | 123.9681 | 94.7166 | 56 | 14 |
| 13 | N-Formylanthranilic acid | Negative | 164.0319 | 91.7858 | 42 | 18 |
|  |  |  | 164.0319 | 120.0136 | 42 | 20 |
|  |  |  | 164.0319 | 135.688 | 42 | 12 |
| 14 | p-Cresol sulfate | Negative | 187.0319 | 79.8117 | 44 | 16 |
|  |  |  | 187.0319 | 106.7375 | 44 | 20 |
| 15 | 3-Indoxyl sulfate | Negative | 212.0319 | 76.8182 | 58 | 34 |
|  |  |  | 212.0319 | 79.8687 | 58 | 20 |
|  |  |  | 212.0319 | 103.7365 | 58 | 26 |
|  |  |  | 212.0319 | 131.7548 | 58 | 18 |
| 16 | p-Cresol glucuronide | Negative | 283.0366 | 84.7512 | 12 | 18 |
|  |  |  | 283.0366 | 106.7905 | 12 | 34 |
|  |  |  | 283.0366 | 112.7071 | 12 | 14 |
|  |  |  | 283.0366 | 174.6041 | 12 | 10 |

**Table S3. Literature reported renal clearance (CL_R_) values of metabolites.**

| **Metabolite** | **Renal clearance (mL/min)** | **N** | **Reference/Comment** |
| --- | --- | --- | --- |
| **Creatinine** | 131.1 | 12 | Miyake 2021^1^ |
|  | 126.3 | 12 |  |
|  | 159 | 14 | Bergagnini-Kolev 2017^2^ |
|  | 105 | 27 | Granda 2024^3^ |
|  | 97 | 17 | Coburn 2002^4^ |
|  | **119.5** |  | **Weighted average** |
| **Pyridoxic acid** | 227 | 14 | Shen 2019^5^ |
|  | 222 | 14 |  |
|  | 328.3 | 6 | Willemin 2021^6^ |
|  | 230 | 23 | Thakur 2025^7^ |
|  | 232 | 17 | Coburn 2002^4^ |
|  | **236.35** |  | **Weighted average** |
| **GCDCA-S** | 6.16 | 6 | Willemin 2021^6^ |
|  | 3.62 | 6 | Tsuruya 2016^8^ |
|  | **4.89** |  | **Weighted average** |
| **Kynurenic acid** | 226 | 27 | Granda 2024^3^ |
|  | 168 | 14 | Tang 2021^9^ |
|  | **206.2** |  | **Weighted average** |
| **p-Cresol sulfate** | 17 |  | Rivara 2017^10^ |
| **p-Cresol glucuronide** | 267.75 |  | Poesen 2016^11^ |
| **3-Indoxyl sulfate** | 67 |  | Rivara 2017^10^ |
| **Taurine** | 8.89 | 6 | Willemin 2021^6^ |
|  | 26.04 | 6 | Tsuruya 2016^8^ |
|  | **17.47** |  | **Weighted average** |

GCDCA-S: glycochenodeoxycholate-3-sulfate

**Table S4. Geometric means (90% confidence interval) of plasma concentration ratios of other OAT1/3 biomarkers in pregnant women (n=46) during late second to early third trimester (T2-T3) and postpartum (PP).**

| **#** | **Metabolite** | **Plasma concentration ratio (T2-T3/PP)** |
| --- | --- | --- |
| 1. | 1-Methyluric acid | 0.39 (0.3-0.5) |
| 2. | 2,6-Dihydroxybenzoic acid | 0.4 (0.33-0.48) |
| 3. | 3-(4-Hydroxyphenyl) lactic acid | 0.68 (0.64-0.73) |
| 4. | 3-Acetylphenol sulfate | 0.5 (0.35-0.71) |
| 5. | 3-Hydroxy-2-methylpyridine sulfate | 0.46 (0.29-0.72) |
| 6. | 3-Methoxycatechol sulfate (2) | 0.58 (0.46-0.73) |
| 7. | 4-Ethylcatechol sulfate | 0.41 (0.29-0.58) |
| 8. | 4-Hydroxyphenylpyruvic acid | 0.77 (0.69-0.85) |
| 9. | 4-Methoxyphenol sulfate | 0.62 (0.48-0.82) |
| 10. | 4-Methylcatechol sulfate | 0.65 (0.55-0.77) |
| 11. | 4-Methylguaiacol sulfate | 0.56 (0.43-0.73) |
| 12. | 5-Hydroxy-2-methylpyridine sulfate | 0.49 (0.29-0.82) |
| 13. | 6-Hydroxyindole sulfate | 0.51 (0.45-0.57) |
| 14. | Androstenediol (3beta,17beta) monosulfate (1) | 0.16 (0.15-0.18) |
| 15. | Androsterone glucuronide | 0.58 (0.49-0.67) |
| 16. | Dihydrocaffeate sulfate (2) | 0.51 (0.4-0.65) |
| 17. | Etiocholanolone glucuronide | 0.41 (0.36-0.47) |
| 18. | Gentisic acid | 0.67 (0.57-0.78) |
| 19. | Glucuronide of piperine metabolite C17H21NO3 (4) | 0.49 (0.39-0.62) |
| 20. | Glucuronide of piperine metabolite C17H21NO3 (5) | 0.51 (0.4-0.64) |
| 21. | Glutamine conjugate of C6H10O2 (2) | 0.41 (0.31-0.53) |
| 22. | Glycine conjugate of C10H14O2 (1) | 0.52 (0.45-0.59) |
| 23. | Glycolithocholate sulfate | 0.53 (0.42-0.68) |
| 24. | Glycoursodeoxycholic acid | 0.3 (0.23-0.38) |
| 25. | Guaiacol sulfate | 0.46 (0.39-0.55) |
| 26. | Indoleacetic acid | 0.5 (0.45-0.56) |
| 27. | Indoleacetylglutamine | 0.44 (0.32-0.6) |
| 28. | Indolin-2-one | 0.42 (0.37-0.47) |
| 29. | N-Acetyl-2-aminooctanoic acid | 0.57 (0.49-0.66) |
| 30. | Phenol sulfate | 0.39 (0.33-0.47) |
| 31. | Sulfate of piperine metabolite C16H19NO3 (2) | 0.69 (0.55-0.86) |
| 32. | Sulfate of piperine metabolite C16H19NO3 (3) | 0.74 (0.59-0.92) |

PP: postpartum; and T2-T3: late second to early third trimester.

**Table S5. Geometric means (90% confidence interval) of fraction unbound in plasma (f_u,plasma_) of OAT1/3 biomarkers (n=12) in late second to early third trimester (T2-T3) and postpartum (PP).**

| **Metabolite** | **f_u,plasma_ (T2-T3)** | **f_u,plasma_ (PP)** | **Ratio (T2-T3/PP)** |
| --- | --- | --- | --- |
| Pyridoxic acid | 0.21 (0.2-0.22) | 0.19 (0.15-0.22) | 1.14 (0.96-1.36) |
| GCDCA-S | 0.0068  (0.0052-0.0089) | 0.0066  (0.0056-0.0077) | 1.03 (0.83-1.29) |
| Kynurenic acid | 0.15 (0.14-0.16) | 0.14 (0.12-0.15) | 1.07 (0.92-1.23) |
| p-Cresol sulfate | 0.03 (0.025-0.035) | 0.026 (0.019-0.034) | 1.16 (0.96-1.41) |
| 3-Indoxyl sulfate | 0.082 (0.071-0.095) | 0.07 (0.066-0.075) | 1.16 (0.98-1.39) |
| Kynurenine | 0.88 (0.83-0.94) | 0.86 (0.8-0.92) | 1.03 (0.94-1.13) |
| N-Formylanthranilic acid | 0.099 (0.083-0.117) | 0.094 (0.087-0.101) | 1.05 (0.91-1.22) |

f_u,plasma_ was calculated using equation 4. Phenylacetylglutamine and p-cresol glucuronide were minimally bound to plasma proteins (f_u,plasma_ ≈ 1). The f_u,plasma_ of taurine could not be calculated due to lack of assay sensitivity. f_u,plasma_: fraction unbound in plasma; GCDCA-S: glycochenodeoxycholate-3-sulfate; PP: postpartum; and T2-T3: late second to early third trimester.

**Table S6. Geometric means (90% confidence interval) of net unbound secretory clearance (CL_sec,u_) of established and putative OAT1/3 biomarkers in pregnant women (n=12) during late second to early third trimester (T2-T3) and postpartum (PP).**

| **Metabolite** | **Estimated net CL_sec,u_ (mL/min)** | | **Net CL_sec,u_ ratio (T2-T3/PP)** |
| --- | --- | --- | --- |
|  | **T2-T3** | **PP** |  |
| **Pyridoxic acid** | 1194 (945-1508) | 1015 (750-1376) | 1.2 (0.9-1.6) |
| **GCDCA-S** | 3442 (2197-5394) | 432 (291-640) | 8 (4.4-14.4) |
| **Kynurenic acid** | 878 (443-1739) | 867 (375-2003) | 1.01 (0.6-1.8) |
| **p-Cresol sulfate** | 695 (534-905) | 497 (349-709) | 1.4 (1.04-1.9) |
| **p-Cresol glucuronide** | 188 (141-251) | 137 (111-170) | 1.4 (1.1-1.7) |
| **3-Indoxyl sulfate** | 1006 (813-1243) | 730 (627-850) | 1.4 (1.1-1.7) |

Net CL_sec,u_ values were calculated using equations 4-5. Net CL_sec,u_: unbound renal secretory clearance; GCDCA-S: glycochenodeoxycholate-3-sulfate; PP: postpartum; and T2-T3: late second to early third trimester.

**Table S7. Clearance values (in mL/min) of OAT1/3 substrate drugs in second and third trimester (T2-T3) and postpartum (PP).**

| **Drug** | **f_e_** | **F** | **f_u,plasma_** | **T2-T3** | | | **PP** | | | **Ratios** | | | **Reference** | |
| --- | --- | --- | --- | --- | --- | --- | --- | --- | --- | --- | --- | --- | --- | --- |
|  |  |  |  | **CL_R_** | **CL_sec_** | **CL_sec,u_** | **CL_R_** | **CL_sec_** | **CL_sec,u_** | **CL_R_** | **CL_sec_** | **CL_sec,u_** |  |  |
| **Amoxicillin** | 0.58 | 0.93 | 0.85 | 411.5 | 276.1 | 324.9 | 257.0 | 175.3 | 206.2 | 1.6 | 1.6 | 1.6 | Andrew 2007^12^ |  |
| **Cefazolin*** | 0.80 | 1.00 | 0.18 | 293.5 | 267.2 | 1484.6 | 170.8 | 149.8 | 832.1 | 1.7 | 1.8 | 1.8 | Philipson 1987^13^ |  |
| **Oseltamivir carboxylate*^#^** | 0.93 | 0.79 | 0.97 | 391.6 | 249.9 | 257.7 | 281.6 | 168.1 | 173.3 | 1.4 | 1.5 | 1.5 | Pillai 2015^14^ |  |
| **Pravastatin** | 0.45 | 0.18 | 0.47 | 566.7 | 475.7 | 1012.2 | 383.3 | 325.0 | 691.5 | 1.5 | 1.5 | 1.5 | Costantine 2016^15^ |  |
| **Tenofovir*** | 0.81 | 0.25 | 0.99 | 197.3 | 52.8 | 53.3 | 155.3 | 39.4 | 39.8 | 1.3 | 1.3 | 1.3 | Best 2015^16^ |  |

*****Since CL_R_ was not reported, it was calculated using the reported oral clearance, f_e_ and F values. **^#^**T2-T3 CL values were compared with CL values in non-pregnant individuals. CL_sec_ was calculated using equation 4. Geometric mean creatinine clearance values (Table 1) in T2-T3 and PP were used to estimate drug filtration clearance. CL_R_: renal clearance; CL_sec_: renal secretory clearance; CL_sec,u_: unbound renal secretory clearance; F: bioavailability; f_e_: fraction excreted unchanged in urine; f_u,plasma_: fraction unbound in plasma; PP: postpartum; and T2-T3: late second to early third trimesters.

**SUPPLEMENTARY FIGURES**

**
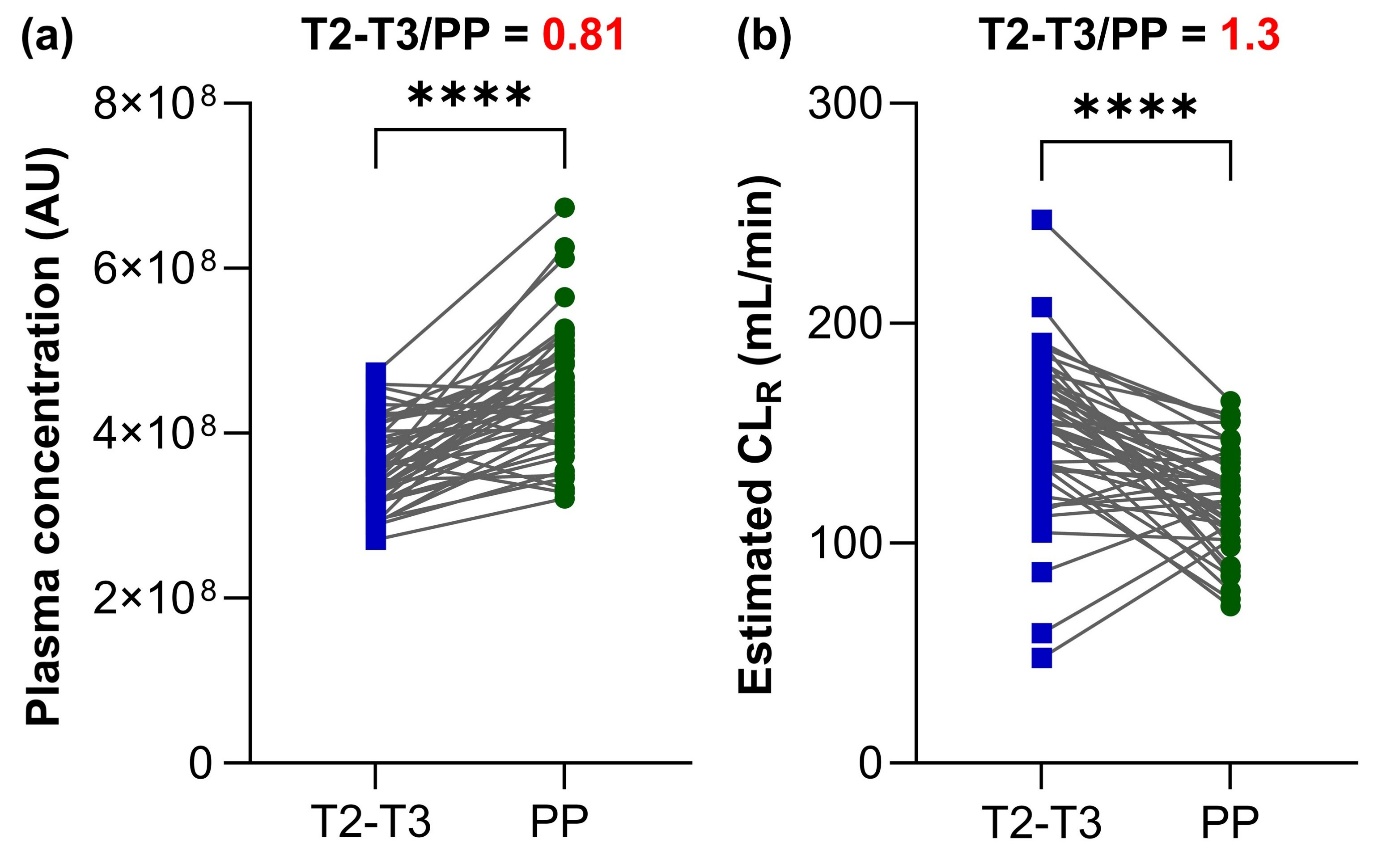
**

**Figure S1. Effect of late second to early third trimester on plasma concentration and estimated renal clearance (CL_R_) of creatinine.** Line and symbol plots of creatinine (a) plasma concentrations and (b) estimated CL_R_ in pregnant women (n=46) in late second to early third trimester (blue squares, T2-T3) and postpartum (green circles, PP). Plasma concentrations and CL_R_ values were compared using the paired t-test. p-value < 0.0001 (****). T2-T3/PP ratios are geometric means (n=46). AU: arbitrary units; PP: postpartum; and T2-T3: late second to early third trimester**.**

**
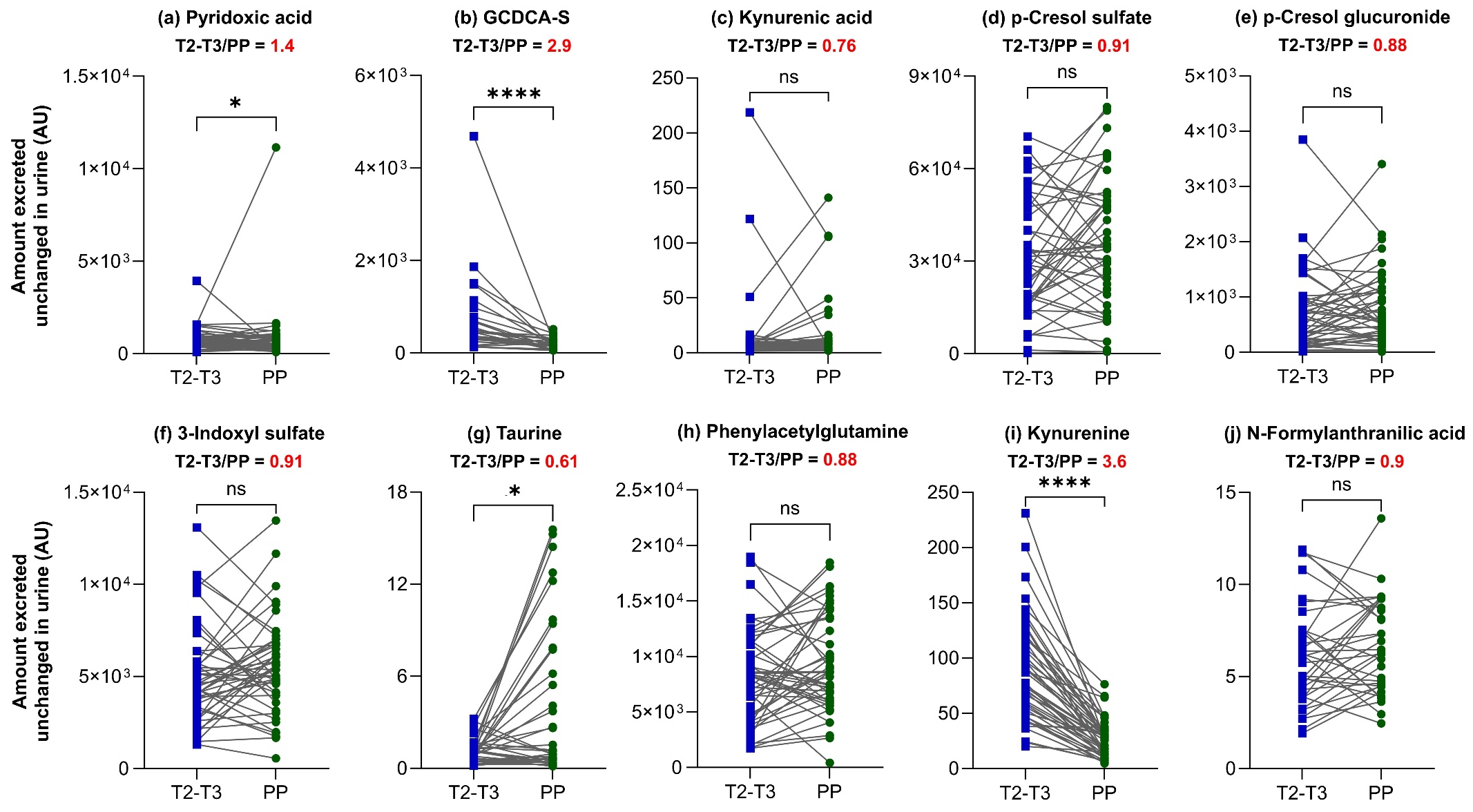
**

**Figure S2. Effect of late second to early third trimester on the amounts of OAT1/3 biomarkers excreted unchanged in urine.** Line and symbol plots of amounts excreted unchanged in urine of (a-j) OAT1/3 biomarkers in pregnant women (n=46) in late second to early third trimester (blue squares, T2-T3) and postpartum (green circles, PP). Amounts excreted unchanged in the urine were compared using paired t-test. p-value < 0.05 (*), and < 0.0001 (****). T2-T3/PP ratios are geometric means (n=46). AU: arbitrary units; GCDCA-S: glycochenodeoxycholate-3-sulfate; ns: non-significant; PP: postpartum; and T2-T3: late second to early third trimester.

**
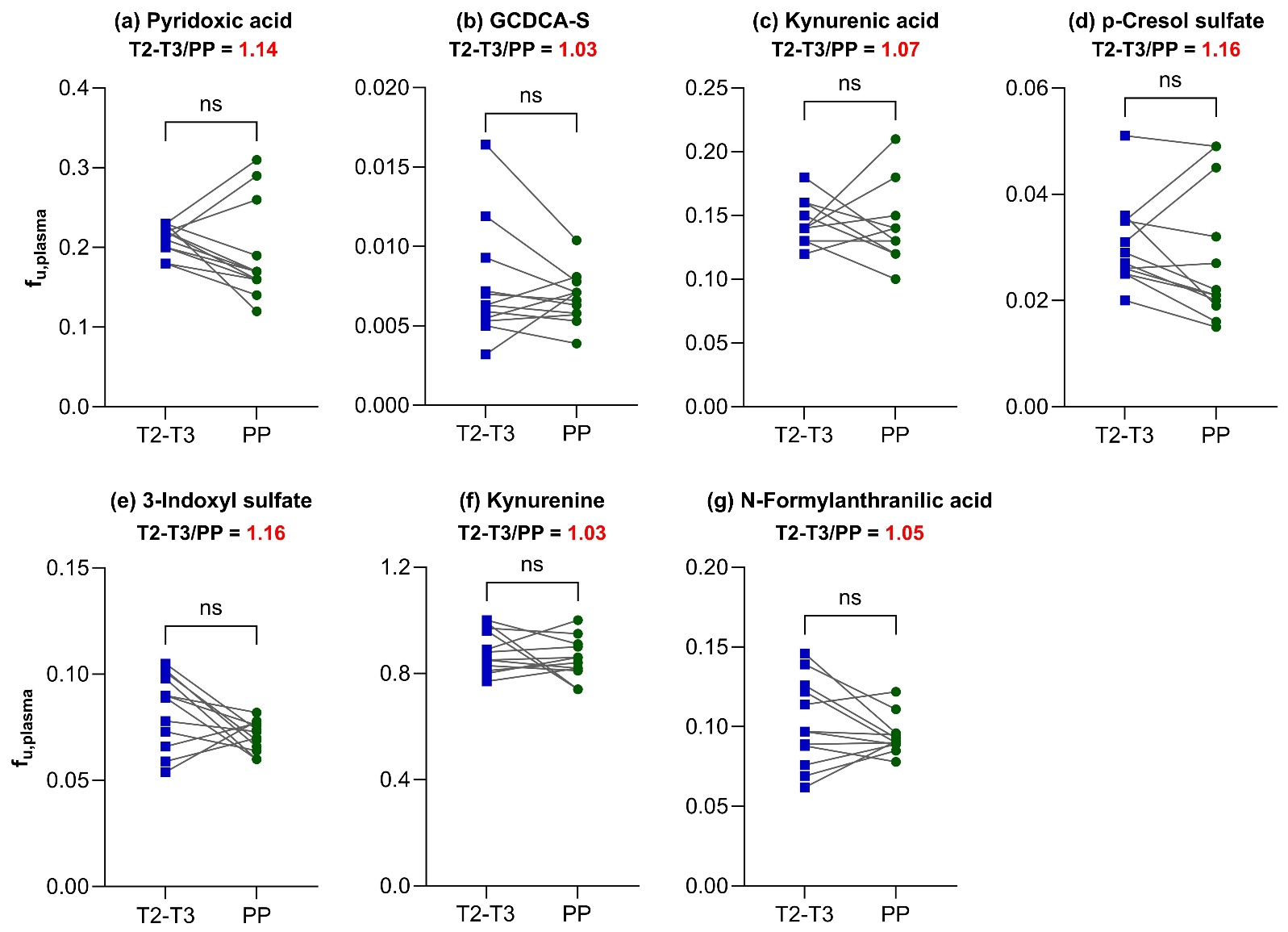
**

**Figure S3. Effect of late second to early third trimester on the fraction of OAT1/3 biomarkers unbound in plasma (f_u,plasma_).** Line and symbol plots of fraction unbound in plasma (f_u,plasma_) of (a-g) OAT1/3 biomarkers in 12 pregnant women randomly selected from the 46 study participants, in late second to early third trimester (blue squares, T2-T3) and postpartum (green circles, PP). f_u,plasma_ was determined using the Thermo RED kit. Data points are arithmetic mean of experimental duplicates and were compared using the paired t-test. T2-T3/PP ratios are geometric means (n=12). GCDCA-S: glycochenodeoxycholate-3-sulfate; ns: non-significant; PP: postpartum; and T2-T3: late second to early third trimester.

**
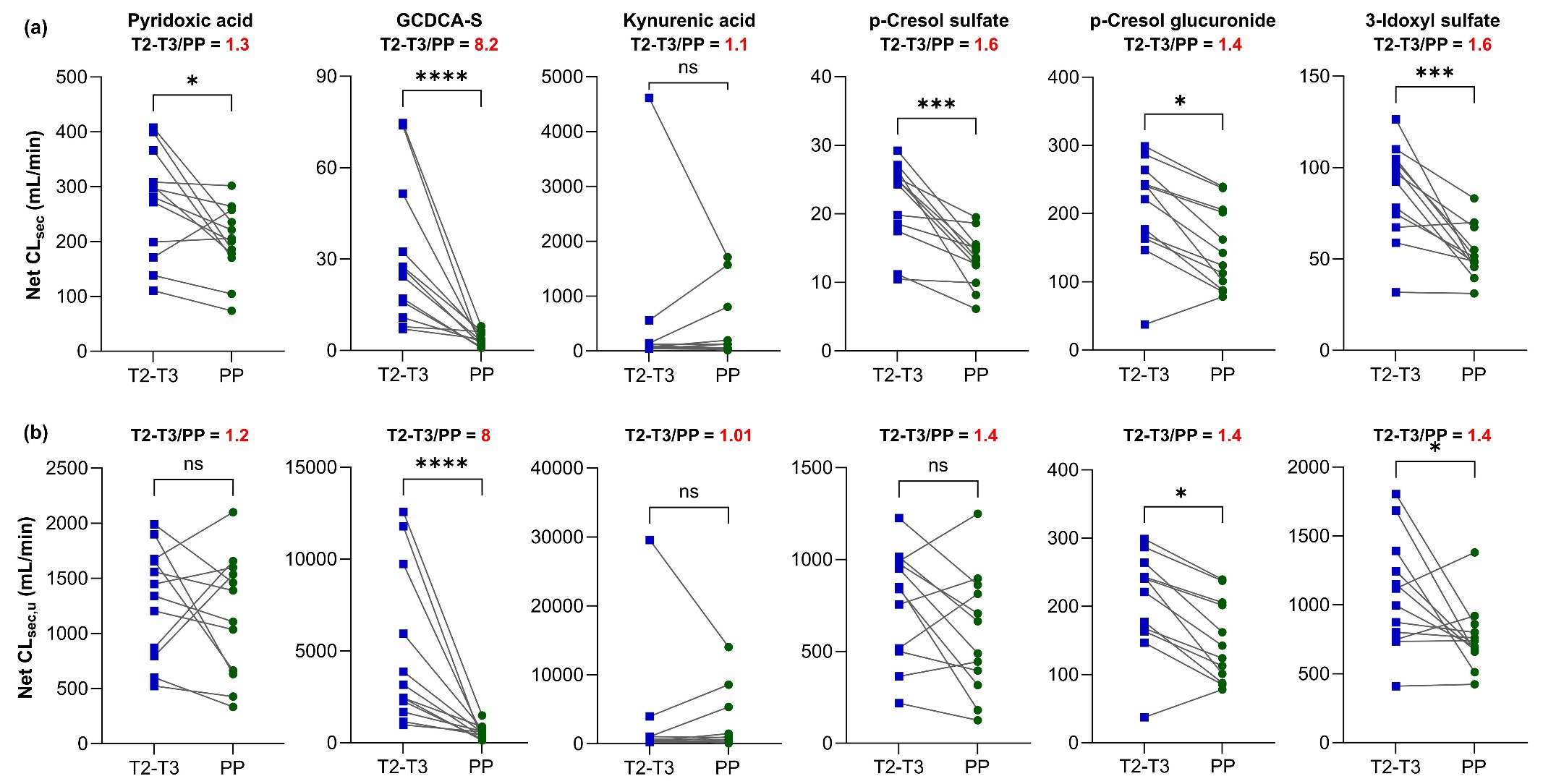
**

**Figure S4. Effect of late second to early third trimester on the net secretory clearance (CL_sec_) and net unbound CL_sec_ (CL_sec,u_) of OAT1/3 biomarkers.** Line and symbol plots of (a) net secretory clearance (CL_sec_, n=12); and (b) net unbound CL_sec_ (CL_sec,u_, n=12) of OAT1/3 biomarkers in pregnant women, in late second to early third trimester (blue square, T2-T3) and postpartum (green circles, PP). Net CL_sec_ and net CL_sec,u_ values were compared using the paired t-test. p-value < 0.05 (*),< 0.001 (***), and < 0.0001 (****). Net CL_sec_ values were calculated using equation 5. T2-T3/PP ratios are geometric means (n=12). GCDCA-S: glycochenodeoxycholate-3-sulfate; ns: non-significant; PP: postpartum; and T2-T3: late second to early third trimester.

**
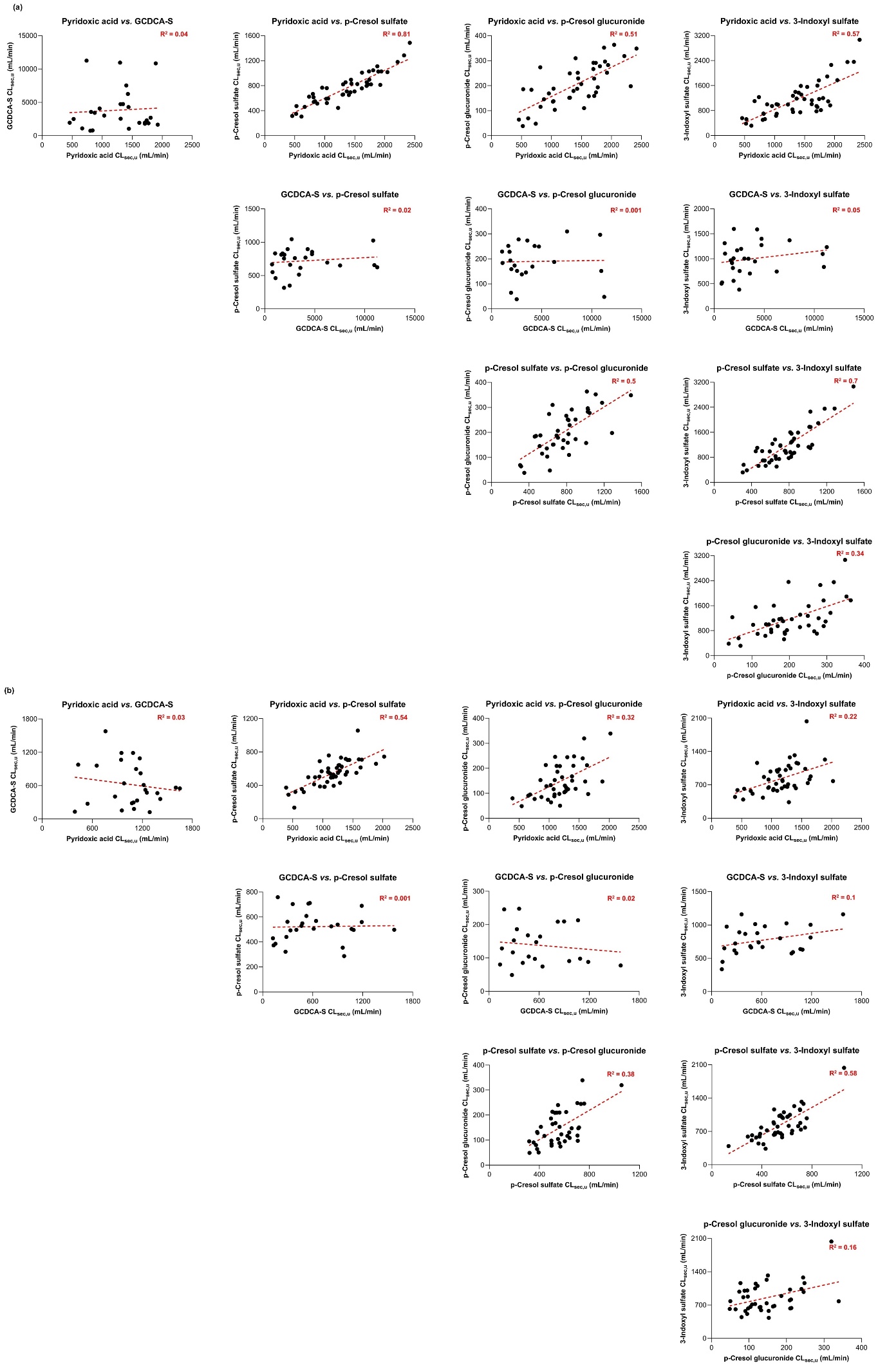
**

**Figure S5. Correlations of net unbound secretory clearance (CL_sec,u_) of OAT1/3 biomarkers in late second to early third trimester and postpartum.** Correlations of net unbound secretory clearance (CL_sec,u_) of OAT1/3 biomarkers in pregnant women (n=46) in (a) late second to early third trimester and (b) postpartum. R^2^ values denote Pearson correlation.

**REFERENCES**

1. Miyake, T. *et al.* Identification of appropriate endogenous biomarker for risk assessment of multidrug and toxin extrusion protein‐mediated drug‐drug interactions in healthy volunteers. *Clin Pharmacol Ther* 109, 507–516 (2021).

2. Bergagnini-Kolev, M. C., Hebert, M. F., Easterling, T. R. & Lin, Y. S. Pregnancy increases the renal secretion of N1-methylnicotinamide, an endogenous probe for renal cation transporters, in patients prescribed metformin. *Drug Metab Dispos* 45, 325–329 (2017).

3. Granda, M. L., Huang, W., Yeung, C. K., Isoherranen, N. & Kestenbaum, B. Predicting complex kidney drug handling using a physiologically-based pharmacokinetic model informed by biomarker-estimated secretory clearance and blood flow. *Clin Transl Sci* 17, e13678 (2024).

4. Coburn, S. P. *et al.* Elevated plasma 4-pyridoxic acid in renal insufficiency. *Am J Clin Nutr* 75, 57–64 (2002).

5. Shen, H. *et al.* Evidence for the validity of pyridoxic acid (PDA) as a plasma-based endogenous probe for OAT1 and OAT3 function in healthy subjects. *J Pharmacol Exp Ther* 368, 136–145 (2019).

6. Willemin, M. E. *et al.* Clinical investigation on endogenous biomarkers to predict strong OAT-mediated drug-drug interactions. *Clin Pharmacokinet* 60, 1187–1199 (2021).

7. Thakur, A., Mathialagan, S., Kimoto, E. & Varma, M. V. S. Pyridoxic Acid as Endogenous Biomarker of Renal Organic Anion Transporter Activity: Population Variability and Mechanistic Modeling to Predict Drug-Drug Interactions. *CPT Pharmacomet Syst Pharmacol* 14, 904–917 (2025).

8. Tsuruya, Y. *et al.* Investigation of endogenous compounds applicable to drug-drug interaction studies involving the renal organic anion transporters, OAT1 and OAT3, in humans. *Drug Metab Dispos* 44, 1825–1933 (2016).

9. Tang, J. *et al.* Endogenous plasma kynurenic acid in human: A newly discovered biomarker for drug-drug interactions involving organic anion transporter 1 and 3 inhibition. *Drug Metab Dispos* 49, 1063–1069 (2021).

10. Rivara, M. B. *et al.* Diurnal and Long-term Variation in Plasma Concentrations and Renal Clearances of Circulating Markers of Kidney Proximal Tubular Secretion. *Clin Chem* 63, 915–923 (2017).

11. Poesen, R. *et al.* Metabolism, Protein Binding, and Renal Clearance of Microbiota–Derived p-Cresol in Patients with CKD. *Clin J Am Soc Nephrol* 11, 1136–1144 (2016).

12. Andrew, M. A. *et al.* Amoxicillin pharmacokinetics in pregnant women: modeling and simulations of dosage strategies. *Clin Pharmacol Ther* 81, 547–56 (2007).

13. Philipson, A., Stiernstedt, G. & Ehrnebo, M. Comparison of the pharmacokinetics of cephradine and cefazolin in pregnant and non-pregnant women. *Clin Pharmacokinet* 12, 136–44 (1987).

14. Pillai, V. C. *et al.* Population pharmacokinetics of oseltamivir in non-pregnant and pregnant women. *Br J Clin Pharmacol* 80, 1042–50 (2015).

15. Costantine, M. M. *et al.* Safety and pharmacokinetics of pravastatin used for the prevention of preeclampsia in high-risk pregnant women: a pilot randomized controlled trial. *Am J Obstet Gynecol* 214, 720.e1-720.e17 (2016).

16. Best, B. M. *et al.* Pharmacokinetics of tenofovir during pregnancy and postpartum. *HIV Med* 16, 502–11 (2015).
